# Generative AI voting: fair collective choice is resilient to LLM biases and inconsistencies

**DOI:** 10.1140/epjds/s13688-025-00612-3

**Published:** 2026-02-09

**Authors:** Srijoni Majumdar, Edith Elkind, Evangelos Pournaras

**Affiliations:** 1 School of Computer Science, University of Leeds, Leeds, LS29JT, UK; 2 Department of Computer Science, Northwestern University, Evanston, IL 60208 US

**Keywords:** Voting, Generative AI, Large language model, Collective decision making, Social choice, Proportional representation, Participatory budgeting, Turnout

## Abstract

Recent breakthroughs in generative artificial intelligence (AI) and large language models (LLMs) unravel new capabilities for AI personal assistants to overcome cognitive bandwidth limitations of humans, providing decision support or even direct representation of abstained human voters at large scale. However, the quality of this representation and what underlying biases manifest when delegating collective decision making to LLMs is an alarming and timely challenge to tackle. By rigorously emulating more than >50K LLM voting personas in 363 real-world voting elections, we disentangle how AI-generated choices differ from human choices and how this affects collective decision outcomes. Complex preferential ballot formats show significant inconsistencies compared to simpler majoritarian elections, which demonstrate higher consistency. Strikingly, proportional ballot aggregation methods such as equal shares prove to be a win-win: fairer voting outcomes for humans and fairer AI representation, especially for voters likely to abstain. This novel underlying relationship proves paramount for building democratic resilience in scenarios of low voters turnout by voter fatigue: abstained voters are mitigated via AI representatives that recover representative and fair voting outcomes. These interdisciplinary insights provide decision support to policymakers and citizens for developing safeguards and policies for risks of using AI in democratic innovations.

**Supplementary Information:**

The online version contains supplementary material available at 10.1140/epjds/s13688-025-00612-3.

## Introduction

Recent advances in artificial intelligence (AI) provide new, unprecedented opportunities for citizens to scale up participation in digital democracy [1–3]. Generative AI in particular, such as large language models (LLMs), has the potential to overcome human cognitive bandwidth limitations and digitally assist citizens to deliberate and decide about public matters at scale [4–8]. This is by articulating, summarizing and even providing syntheses of complex opinions [7, 9, 10], with a potential to mitigate for the voter fatigue and reduced voter turnout [10–12], while fostering common ground for compromises, consensus and lower polarization [4, 10, 11, 13, 14]. However, understanding the implications and risks of using large language models for decision support, recommendations or even direct representation of human voters is a pressing challenge [15–17].

**Figure 1:**
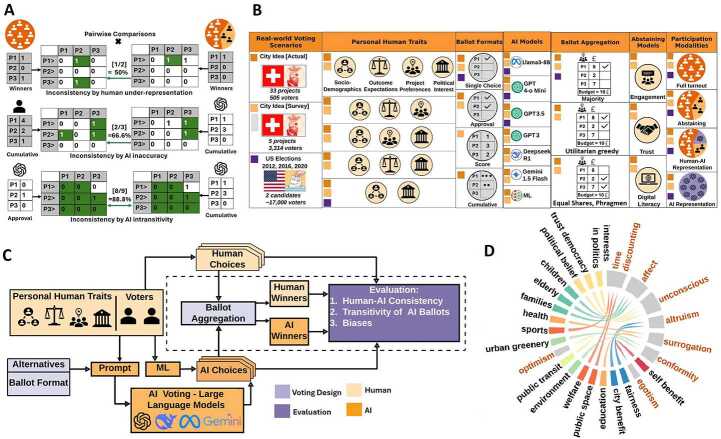
An overview of the studied generative AI voting framework. (A) Three manifestations of choice inconsistency are distinguished, measured using Condorcet pairwise matches: (i) inconsistencies by under-representation as a result of low voters turnout, (ii) inconsistencies by inaccuracy of AI choice to approximate human choice and (iii) inconsistency by intransitivity of AI choices over different ballot formats. P1, P2 and P3 are projects put up for voting and received the scores 4, 2 and 1, respectively, by a voter; in the case of approval voting, the scores are 0 or 1. (B) The factorial design with the 7 studied dimensions: (i) Real-world voting scenarios in the context of participatory budgeting and national elections. (ii) Various combinations of personal human traits (features), based on which AI voting personas are created. (iii) Four ballot formats. (iv) Seven AI models, six large language models and a predictive machine learning model (benchmark). (v) Ballot aggregation methods for elections and participatory budgeting. (vi) The three abstaining models that are based on engagement, digital literacy and trust. (vii) Participation modalities ranging from exclusive human participation of varying turnout to mixed populations of humans and AI representatives of abstained voters. The studied combinations for each voting scenario are marked with different colors, see also Table 1. (C) The framework of generative AI voting. For each voter in the real-world voting scenario, a prompt is given to large language models to construct the voting persona. The input is the personal human traits, the voting options and the ballot format, with instructions for the voting persona on how to make a choice. This choice is the output of the persona. Both human and AI choices are aggregated using a ballot aggregation method. The inconsistencies of individual and collective choices for humans and AI personas are assessed, along with potential biases that explain these inconsistencies. (D) The personal human traits are mapped to cognitive biases. Section S3.1 illustrates the origin of choice inconsistencies to potential cognitive biases.

**Unraveling inconsistencies in generative AI voting.** We disentangle the inconsistencies of large language models when employed to generate *individual voter choices* and assess the ways in which these inconsistencies shape the *collective choice*. In particular, we study three manifestations of choice inconsistency as shown in Fig. 1a:

1. **Inconsistency in voting outcomes by under-representation due to low human voters turnout**.
  It is measured by the dissimilarity in collective choices when voters abstain compared to when they participate;
2. **Inconsistency by inaccurate approximation of human choice by AI**.
  It is measured by the dissimilarity between AI and human choices, and
3. **Inconsistency by intransitivity** [18, 19] **of AI choice**.
  It is measured by the dissimilarity in AI choices across different ballot formats.

Since intransitivity is also present in human choices, particularly in polarized contexts [19] that are often shaped by biases [20, 21], it is reasonable to expect similar inconsistencies to appear in LLM choices. Whether potential biases that explain the inconsistencies between human and AI choices are of a different nature than the ones between different input voting methods is an open question studied in this article. We rigorously measure such inconsistencies with a single universal approach grounded in social choice theory [13, 22]. It exhaustively characterizes the similarity of the two choices (individual or collective) by counting the relative number of Condorcet pairwise matches; see Section 4.2 for further information. We also explore causal links of these inconsistencies to potential cognitive biases triggered by the input to large language models, based on which choices are made.

**Generative AI voting: a converging technological advance with inevitable challenges.** Large language models have been applied to predict election outcomes using sensitive demographic information reflecting the political profile of individuals [23]. They have also been employed to predict pairwise comparisons of proposals for constitutional changes [6] and to facilitate deliberation by summarizing opinions expressed in free-form text [24, 25]. However, little is known about whether this AI predictive capability can expand to voting with complex ballot formats that involve more options to choose from [5]. Participatory budgeting [26] is one such process put under scrutiny in this article. Here city authorities distribute a public budget by letting citizens propose their own project ideas, which they vote for and often implement themselves [27]. Projects may be pertinent to different impact areas (e.g., environment, culture, welfare), beneficiaries (e.g., elderly, children) and can have different costs [28]. Voters can approve, rank, or distribute points over their preferred projects, while winners are elected based on the popularity of the projects (*utilitarian greedy*) or based on a proportional representation of the voters’ preferences (*equal shares* or Phragmen’s rule) [29, 30]. So far, AI assistance for such processes is limited. A participatory budgeting process has been emulated using AI agents to examine the feasibility of consensus building by assisting voters in electing winners through a reinforcement learning framework [31]. This work focuses on promoting compromises using rewards to reach consensus instead of applying a ballot aggregation method. In the context of vote prediction, Yang et al. recently conducted a study in which large language models (LLMs) emulate voters to generate preferences and to examine the diversity of preference generation through a lab experiment involving 180 university students [32]. However, the study does not evaluate the impact of LLM-based voting on real-world participatory processes. It also does not address the influence of voters who are more likely to abstain on voting outcomes. Moreover, the scope and citizens’ engagement in participatory budgeting campaigns remain, to a large extent, a one-shot and rooted in local civic cultures [11, 33]. With such complexity and degree of design freedom, scaling up participatory budgeting turns into the ultimate democratic blueprint to assess capabilities and risks of generative AI voting. We do not make a normative statement about the use of (generative) AI voting, although prominent scholars have explored this plausible future; for instance, Augmented Democracy by Hidalgo et al. [34], along with recent research [23, 32] that explore the potential for scaling up direct citizen participation in decision-making, rather than over-relying on human representatives. This scenario, though, seems highly relevant as a result of an inevitable technological convergence of AI and digital voting, for instance, allowing personal and localized AI assistants to interoperate with the Application Programming Interfaces (APIs) of digital voting platforms. Understanding the implications of such capabilities and preparing safeguards to protect democracy and mitigate the consequences of AI risks comes with merit and urgency, which we address in our work.

**How resilient representative voting outcomes are with generative AI?** We hypothesize that a proportional ballot aggregation method can build up *resilience* for representative voting outcomes if AI representatives are used for human voters who would otherwise abstain or lack the capacity to actively participate (see participation modalities in Fig. 1b). In other words, *we examine whether inconsistencies in collective voting outcomes resulting from low voter turnout (see Fig. **1**a) are greater than those arising from generative AI representatives of abstaining voters using different ballot aggregation methods*. This process of consistency recovery through AI representation indicates the degree to which the original outcome can be preserved. We refer to this as the *resilience* of a voting outcome in scenarios of low voter turnout and mixed populations composed of humans and AI representatives of abstaining voters.

**Disentangling the role of voting design in generative AI voting.** The inconsistencies of generative AI voting, their association with ballot formats and aggregation methods, along with the potential AI and human biases explaining these inconsistencies, are systematically studied here for the first time using a novel factorial design based on real-world empirical evidence. It consists of seven dimensions (see Fig. 1b) designed to emulate AI voting representation, generate individual choices and aggregate them into a collective voting outcome.

1. *Real-world voting scenarios* - election datasets from the 2012, 2016 and 2020 US national elections [35] as well as data from the 2023 participatory budgeting campaign of ‘City Idea’ in Aarau, Switzerland [36] are studied. The latter dataset includes two voting scenarios: a hypothetical one provided to voters before voting via a *survey* and the *actual* voting data. The datasets from Aarau also contain demographic data and personal information traits collected before and after voting through pre-voting and post-voting surveys. This information is used to capture individual voter context when emulating AI representations through prompt engineering in large language models. These three datasets cover a wide range of ballot types (e.g., single choice ballots for US elections and approval or score/cumulative ballots for the Aarau voting), voting alternatives and numbers of voters to experiment with; see Fig. 1b and Section 4.1.
2. *Personal human traits* - for each voter, multiple incremental levels of additional information are provided as input to large language models to generate ballots. This includes (i) socio-demographic characteristics (e.g., gender, age, education, household size), (ii) political interests (e.g., ideological profile, political beliefs), (iii) personal attitudes toward project preferences (e.g., prioritization of green initiatives, sustainable transport, elderly care facilities) and (iv) preferences for the qualities of voting outcomes (e.g., favoring cost-effective winning projects, popular projects, or projects with proportional representation of citizens’ preferences). These traits are obtained from voter feedback surveys, which are linked to actual voting behavior in the Aarau voting scenarios, or collected during voter registration for the US elections (Tables S3–S7). Not all traits are available across all datasets (see the distribution of extracted human traits in Fig. 1b).
3. *Ballot formats* - four methods with incremental levels of complexity and expressiveness are compared [19, 37]. These include single choice for all voting scenarios, n-*approvals* (‘n’ of projects approved), score (assigning a preference score from a specified range [1 to 5] to each option) and cumulative voting (distributing a number of points (i.e., 10) over the options) [38–40] for the participatory budgeting scenarios.
4. *AI models* - generative and predictive AI methods have been used to emulate AI representation. Six large language models [41, 42] are assessed along with a more mainstream predictive machine learning (ML) model used as a benchmark. GPT 4-o Mini, GPT3, GPT3.5, Deepseek R1, Gemini 1.5 Flash and Llama3-8B are chosen, covering a wide spectrum of capabilities in open-source and proprietary generative AI (more details on prompts and choice generation in Section S1.2) [43]. The predictive ML benchmark is built by using personal human traits as features to predict ballots using neural networks (more details in Section S3.3) [44].
5. *Ballot aggregation methods* - majority aggregation is used to determine the collective outcome of the US elections. For the participatory budgeting scenarios, the utilitarian greedy method, the method of equal shares [45] and Phragmén’s sequential rule [46] are employed. *Utilitarian greedy* simply selects the next most popular project, the one with the highest number of votes, provided the available budget is not exhausted. *Equal shares* ensures proportional representation of voters’ preferences by dividing the budget equally among voters as endowments. Voters can only use their share to fund projects they voted for. The method evaluates all project options, starting with those receiving the most votes, and selects a project if it can be funded using the budget shares of its supporters. A full explanation of equal shares is beyond the scope of this article and can be found in earlier work [45, 47, 48]. In practice, equal shares may sacrifice an expensive popular project in favor of several low-cost projects that collectively satisfy more voters’ preferences [28, 29]. Because of this effect, it is likely that consistency measurements based on the pairwise similarity yield higher values for equal shares. This is the reason we control for the number of winning projects in equal shares by counting a subset of the most popular winning projects, which is equal in number to the winners of the utilitarian greedy method. Phragmén’s sequential rule is another proportional aggregation method that balances fairness and representation between groups, in contrast to equal shares, which emphasizes fair representation within groups by ensuring that at least one voter from each group is represented [49]. Equal shares was the method actually used in the City Idea campaign to select winners [28], providing strong realism for the findings of this study.
6. *Abstaining models* - three types of abstaining voters: (i) those with low digital skills, limiting their ability to participate online [12, 50] and often leading to low turnouts [6, 8, 51, 52]; (ii) those with low political engagement [53, 54]; and (iii) those who distrust institutions [52, 55, 56]. Using pre-voting and post-voting survey questions from the City Idea participatory budgeting campaign (Tables S5–S7), we identify proxies for these abstaining profiles [51, 57] and divide voters into quartiles to distinguish voters who are likely to abstain. The share of the population that meet the criteria of the three abstaining models is 36.1%, 48.3% and 27.4% respectively.
7. *Participation modalities* - we assess the consistency of voting scenarios with full and low turnouts of human voters, partial/full AI representation of abstained voters and AI representation of the whole human population.

**Assessing generative AI voting in action.** Voting personas are constructed using input prompts of large language models as depicted in Fig. 1c. This designed process aims to emulate the three voting scenarios with the different settings of Fig. 1b. Each input prompt consists of a standardized description of the voter’s profile (see Section S1) and an instruction to vote according to the ballot format. The consistency between the individual and collective real-world choices of humans and AI personas is compared for the first time by measuring the Condorcet pairwise matches as shown in Fig. 1a [13, 19, 22]. These consistency values are then becoming the dependent variable to predict using the personal human traits as independent variables (features), fed into a neural network (see Section 4.3). Based on a systematic mapping of human personal traits to cognitive biases as illustrated in Fig. 1c (see Section S3.1 for more detail), this prediction model causally explains the human traits that contribute to inconsistencies and the potential underlying biases that explain these inconsistencies. This novel analysis is designed to provide a significant conceptual advance in understanding how voting design reinforces or mitigates different AI biases in real-world practice.

## Results

**Figure 2:**
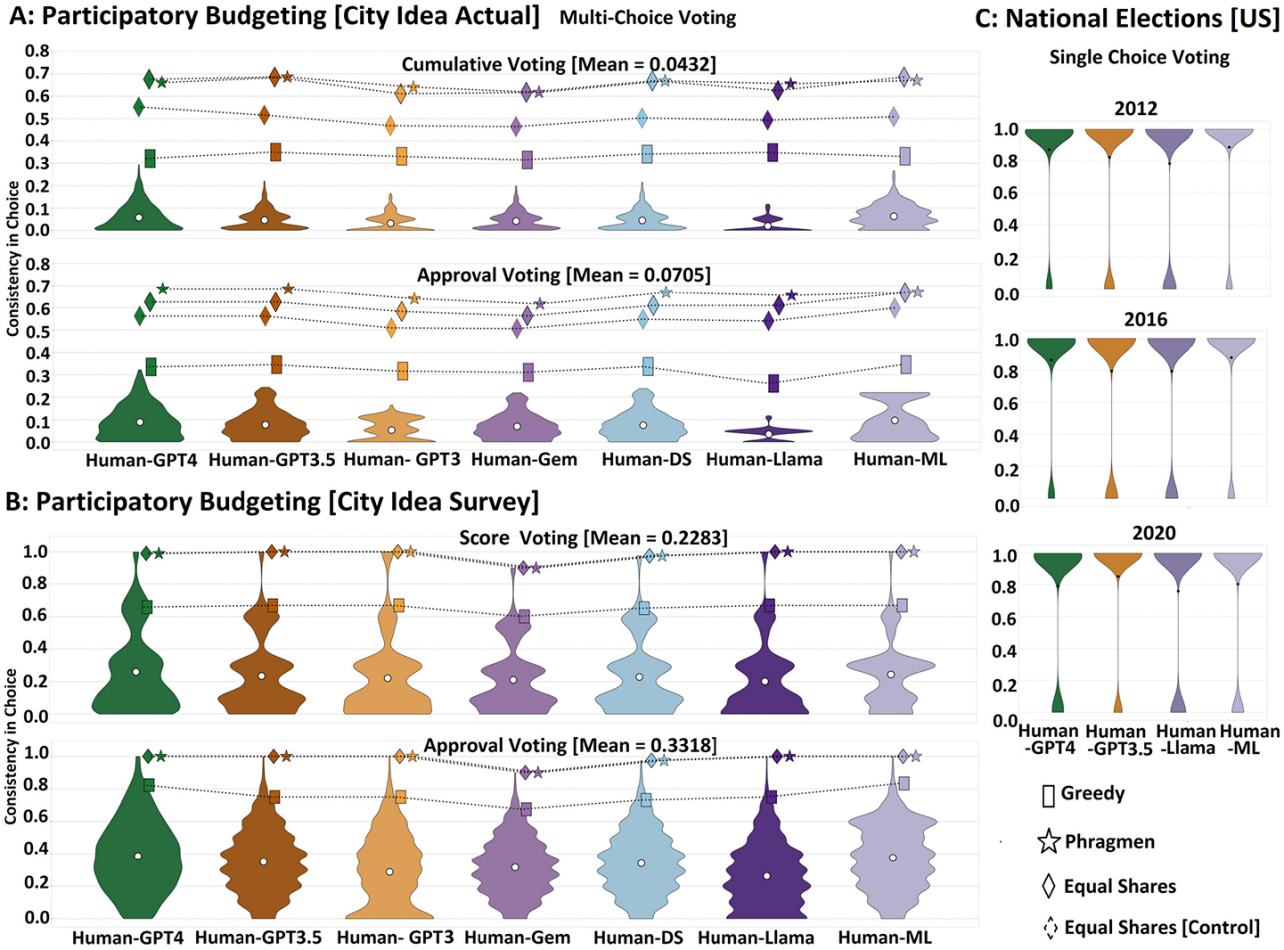
Choice by large language models is consistent with humans for single choice majoritarian elections; however, accuracy drops for more complex ballots with a larger number of alternatives, as in the case of participatory budgeting. Strikingly, the accuracy of collective choice is significantly higher than that of individual choice, particularly for the fairer ballot aggregation rules of equal shares and Phragmén’s. GPT 4-0 Mini shows the highest consistency and Llama3-8B the lowest among the large language models, which, though, remain inferior to a predictive machine learning model. The mean consistency (y-axis) for different population of voters (10%, 25%, 75% and 100%) in individual and collective choice is shown for six large language models (x-axis): GPT 4-o Mini (GPT 4), GPT3.5, GPT3, Gemini 1.5 Flash (Gem), Deepseek R1 (DS) and Llama3-8B (Llama) along with the predictive AI model (*ML*), across three real-world voting scenarios: The participatory budgeting campaign of City Idea, (A) actual and (B) survey, as well as (C) the US national elections of 2012, 2016 and 2020. For participatory budgeting, the ballot formats of cumulative/score (top) and approval (bottom) are shown, including the ballot aggregation methods of equal shares, Phragmén’s, and utilitarian greedy. For the actual voting of City Idea, the accuracy of equal shares is calculated for all winners and a controlled number of winners (as many as utilitarian greedy) for a fairer comparison.

The following three key results are illustrated in this article:

1. Fair voting methods to elect winners are more resilient to inconsistencies of AI to accurately estimate human choice, demonstrating a striking underlying win-win relationship: fairer voting outcomes for humans with fairer human representation by AI (Figs. 2 and 4). These inconsistencies are particularly prominent in complex ballot formats with a large number of alternatives, while simple majoritarian voting tends to be highly consistent. AI intransitivity across ballot formats is higher than that of humans, with a greater impact on collective choice when the number of alternatives is large (Fig. 3).

**Figure 3:**
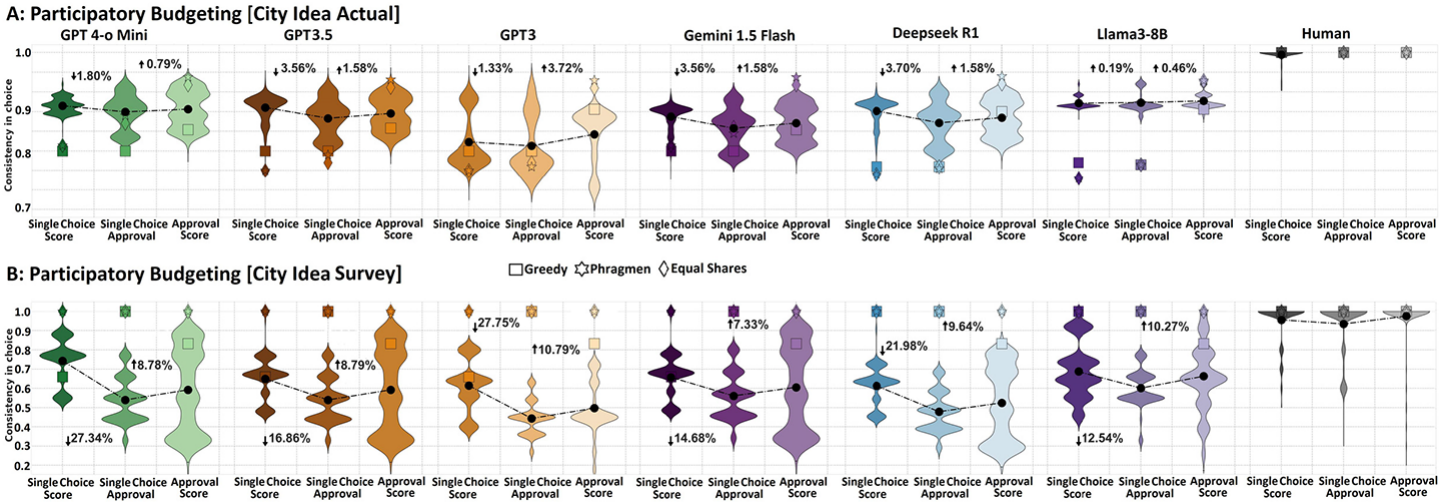
Intransitivity of AI across different pairs of ballot formats is higher than that of humans, which remains negligible. AI intransitivities have a higher influence on the consistency of voting outcomes over a large number of alternatives. Llama3-8B predicts ballots that are not very diverse and select a limited set of projects, which results in higher transitivity compared to other language models. Equal shares and Phragmén’s also show here a higher capacity to mitigate the ballot intransitivities. It achieves more than 80% consistency in preserving voting outcomes between cumulative and approval ballots. The consistency (y-axis) in individual choice among different pairs of ballot formats (x-axis) is shown for six large language models (GPT 4-o Mini, GPT3.5, GPT3, Gemini 1.5 Flash, Deepseek R1 and Llama3-8B), humans and the two voting scenarios in the participatory budgeting campaign of City Idea: (A) actual vs. (B) survey. Mean consistency values are calculated across randomly sampled population of 25%, 50%, and 75%
2. AI representation is more effective for a voter who is likely to abstain than for an arbitrary voter, particularly under fair collective choice (Fig. 4). Abstaining voters result in a representation deficit that is restored by AI, while AI representation over arbitrary voters mainly has a noise-reduction effect on the voting outcome.

**Figure 4:**
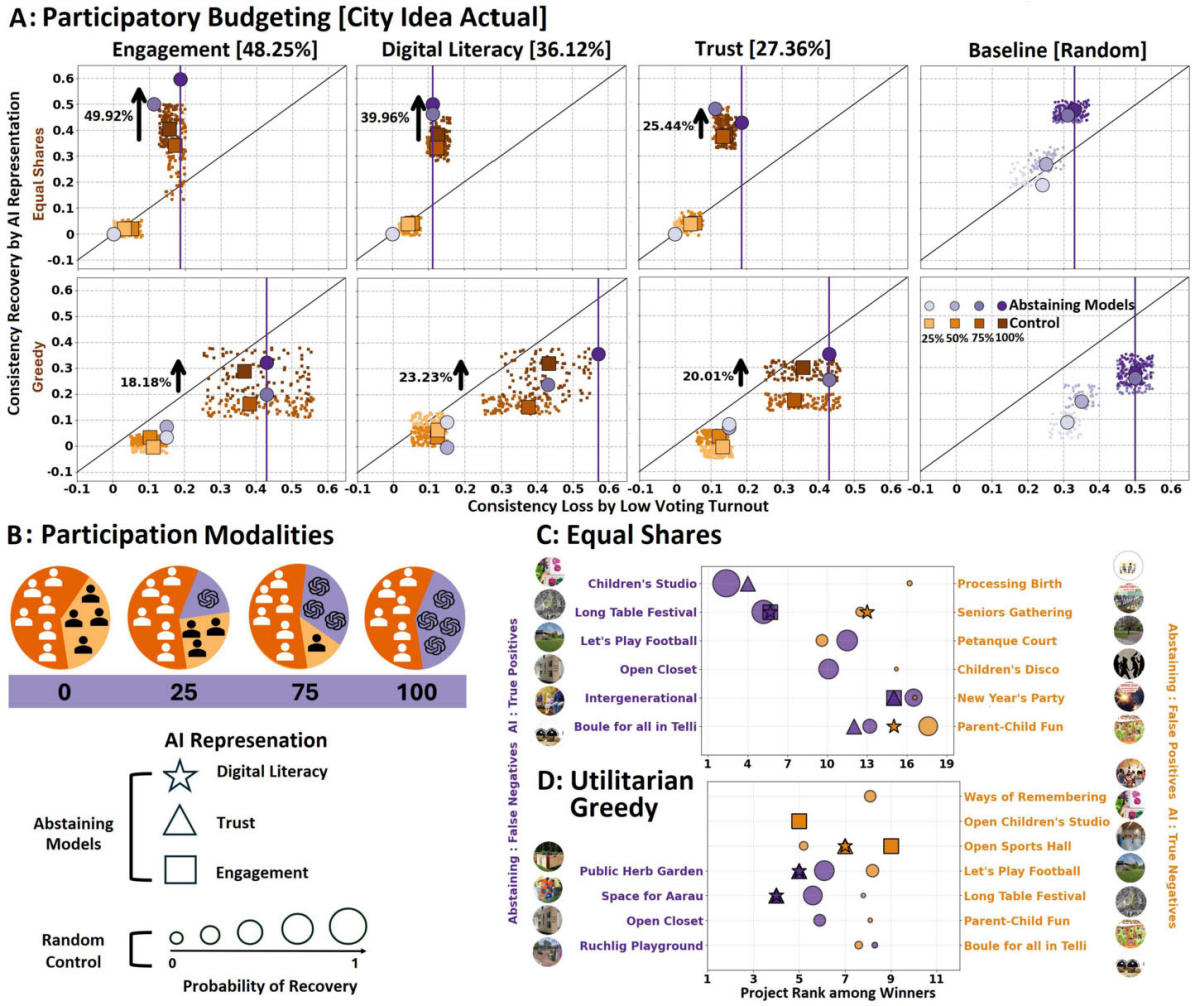
Representing more than half of human abstaining voters with AI results in significant consistency recovery, in particular for fair ballot aggregation methods. Strikingly, AI representation of abstained voters is more effective than representing arbitrary voters (random control). Consistency recovery is at two levels: (i) False negative projects removed under abstaining but added back by AI representatives, which are higher in ranking and number than (ii) false positive projects added under abstaining but removed by AI representatives. The consistency loss in voting outcomes by low voters turnout (x-axis) is emulated by removing different ratios of human voters (25%, 50%, 75% and 100%) among the whole population (baseline) and those who are likely to abstain: low engagement, trust, and digital literacy profile (% of the abstaining populations in the brackets on top). A consistency recovery (y-axis) is hypothesized by AI representation using GPT3.5. (A) Actual participatory budgeting campaign of City Idea. (B) Studied participation modalities. (C)-(D) Depicts which projects are recovered or added by AI representation of abstaining voters (digital literacy, trust, low engagement). When voters abstain, some projects are sacrificed, and purple markers represent the projects added back using AI representation. The orange projects are those that newly emerge as winners with AI representation. The projects and their probability to recover consistency under random control (recovery of 30 groups of random voters, each of size equal to the abstaining voters) are shown for comparison
3. Features of abstaining voters related to their low engagement, digital literacy and trust explain the consistency of their AI representation and the transitivity of ballot formats (Fig. 5). Affect and unconscious biases explain the (in)consistency of human-AI choice, while time-discounting biases explain the transitivity of AI choice across ballot formats.

**Figure 5:**
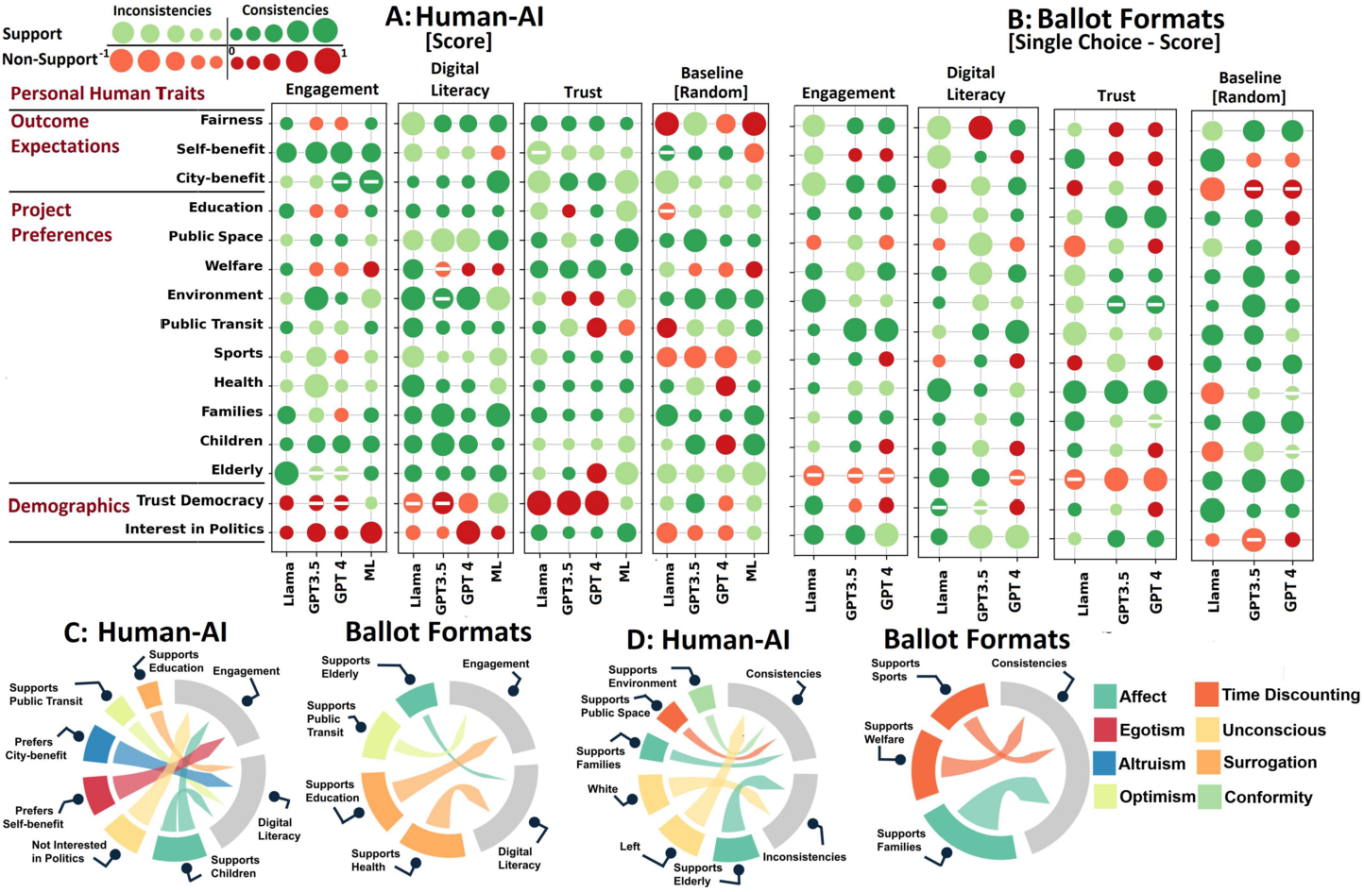
Compared to an arbitrary abstaining voter, those with low engagement and digital literacy exhibit characteristics that explain the consistency of human-AI representation and ballot formats, for instance, no interest in politics and support to education/health projects related to unconscious and surrogation biases. Time discounting, affect and conformity biases, such as preference for public space and environmental projects as well as support to families contribute to the consistency of human-AI choice (shown for one proprietary and one open-source generative AI model along with predictive AI). Time discounting factors such as preference for sport, and welfare projects as well as affect heuristics such as preference for projects that benefit families, explain AI consistency among ballot formats. The relative importance of the personal human traits (y axis) for the actual participatory budgeting campaign of City Idea, using GPT 4-o Mini, GPT3.5, Llama3-8B, and the predictive AI model (*ML*) on the x axis, is depicted by the size of the bubbles and is calculated using Shapley additive explanations. The consistency of human-AI representation for (A) ballot formats (B) (single choice vs. cumulative) is assessed. For each of these, the personal human traits explain the following: (i) The consistency difference between the three abstaining models and their random control. (ii) The (in)consistency of AI representation and transitivity for the whole population. The ‘-’ sign indicates non-significant values (p>0.05). (C)-(D) The statistically significant biases present in all AI models and datasets are summarized by chord diagrams

### Fair collective choice is resilient to human-AI inconsistencies

Voting design and choice context have an impact on human-AI inconsistencies. Fig. 2 illustrates the human-AI consistency in individual and collective choices for single choice and multi-choice voting. For multi-choice voting, the individual and collective consistency of human and AI choices are measured as the average consistency across various sampled population sizes of 25%, 50% and 75% (shown separately in Fig. S2). The consistency of individual choice remains poor in complex ballot formats with several alternatives. On average, it is 5.68% and 28.005% for the actual and survey voting scenarios of City Idea, yet it is 84.5% for the binary majoritarian US elections. GPT 4-o Mini shows the highest consistency of individual choice among the seven proprietary and open-source large language models, which is 4.85% and 7.85% higher than GPT3.5 and Llama3-8B, respectively. We observe that Gemini 1.5 Flash and Deepseek R1 have comparable performance with GPT3.5. On the contrary, the consistency of collective choice increases by 46.78% overall. Strikingly, the consistency of equal shares and Phragmen’s is on average 69.9%, which is 31.6% higher than utilitarian greedy. Even when reducing the number of winners in equal shares to that of utilitarian greedy, the consistency remains 22.8% higher. The consistency differences of the proportional methods compared to utilitarian greedy, without or with the same number of winners, are statistically significant with (p < 0.03) and (p < 0.04), respectively. Compared to large language models, the machine learning model shows 1.7% higher consistency in individual choice and 2.9% higher in collective choice. The consistency values shown here are based on the AI emulations using all personal human traits, as shown in Fig. 1a. Removing project preferences from the context of AI choice generation results in the highest consistency reduction of 18.1%, whereas political interest leads to the lowest reduction of 3.5%.

**Intransitivity: higher for AI with impact on collective choice among many alternatives.** Fig. 3 illustrates the transitivity of preferences in different large language models and humans by measuring the consistency of individual and collective choices across different pairs of ballot formats (see Section 1 and Fig. 1a). While human transitivity averages 97.1%, AI transitivity is 74.3% for GPT 4-o Mini, 72.1% for GPT3.5, 76.2% for Llama3-8B and 71.23% for Gemini 1.5 Flash. In terms of collective choice, equal shares show 12.2% higher consistency than utilitarian greedy (p < 0.04). Equal shares achieve more than 80% consistency among winners based on cumulative, score, and approval ballots. For the actual voting of City Idea, ‘approval-cumulative’ voting demonstrates the highest transitivity, which is 4.4% higher than ‘single choice-approval’ and 3.2% higher than ‘single choice-cumulative’. However, for the survey of City Idea, ‘single choice-cumulative’ shows the highest transitivity, which is 25.2% higher than ‘single choice-approval’ and 15.1% higher than ‘approval-cumulative’ (p < 0.03).

### AI representatives to recover from low voters turnout

**Assessing consistency recovery by AI representatives.** Fig. 4 illustrates the capability of AI representatives to recover the consistency of voting outcomes lost by low voter turnout. For a certain set of projects that are winners in the final voting outcome when all voters participate, abstaining can therefore lead to an outcome with fewer or more projects. The winning projects removed due to the abstaining population represent a loss of consistency in the voting outcome. Consistency recovery using AI representation for voters who are likely to abstain is electing winning projects that contain or remove the projects that would be erroneously removed or added respectively while abstaining. It is calculated as the difference in consistency of the two scenarios, see Section 4.2. The three abstaining models (low engagement, trust, and digital literacy) are assessed along with the baseline that determines random abstaining voters across the whole population. Four participation modalities (Fig. 1a) are studied: (i) Human voters exclusively with 100% of voters’ turnout. (ii) Human voters exclusively with varying turnout levels in the range [25%,100%] with a step of 25%. The maximum number of abstaining voters is either the total voters (baseline in Fig. 4b) or the number of voters with low digital literacy, engagement, and trust as determined by the abstaining models. (iii) Mixed populations of human voters and AI representatives of abstaining voters in the range [25%,100%] with a step of 25%. (iv) AI representatives exclusively. We show the consistency recovery in the actual voting scenario for the City Idea campaign using GPT3.5 in Fig. 4 and using the other large language models (GPT 4-o Mini, Llama3-8B, Gemini 1.5 Flash, GPT3, and Deepseek R1) in Section S2.4. The results on consistency recovery by AI representatives in the survey voting scenario of City Idea have been shown in Fig. S9.

**Can AI representatives mitigate for abstained voters?** Strikingly, up to 75% of AI representation of low-engaged abstaining voters (94 representatives out of 126 abstaining voters in a population of 252 voters, see Section S2.3) is sufficient to recover up to 50% higher lost consistency than the random control population using equal shares. This superior consistency recovery is also observed for abstaining voters with low digital literacy (39.96%) and trust (25.44%). The fair aggregation rules of equal shares and Phragmén’s achieve, on average 7.53% higher recovery compared to utilitarian greedy for all the abstaining models. Even when controlling for the same number of winners, fair ballot aggregation methods achieve higher recovery than utilitarian greedy by 6.72% (p < 0.05). Comparing the different AI models, we earlier observed in Fig. 4 that the collective consistency, that is the one between voting outcomes corresponding to humans and those corresponding to 100% AI representation (Fig. 2), is comparable for GPT 4-o Mini and GPT3.5, with no statistically significant difference. We notice a similar trend here, where AI representation by GPT 4-o Mini achieves 2.1% higher recovery than GPT3.5, which is though not statistically significant (p=0.092) (Figs. S5, S6). However, AI representation by GPT 4-o Mini shows significant differences in consistency recovery compared to Llama3-8B and GPT3, outperforming them by 6.4% and 8.2% respectively (Figs. S6, S7 and S8). GPT3.5 performs better than Llama3-8B and GPT3, achieving recovery gains of 4.61% and 5.97%, respectively (Figs. S6, S7, S8, and Table S11). Strikingly, up to 75% of AI representation of low-engaged abstaining voters (94 representatives out of 126 abstaining voters in a population of 252 voters, see Section S2.3) is sufficient to recover up to 50% higher lost consistency than the random control population using equal shares. This superior consistency recovery is also observed for abstaining voters with low digital literacy (39.96%) and trust (25.44%). The fair aggregation rules of equal shares and Phragmén’s achieve, on average 7.53% higher recovery compared to utilitarian greedy for all the abstaining models. Even when controlling for the same number of winners, fair ballot aggregation methods achieve higher recovery than utilitarian greedy by 6.72% (p < 0.05). Comparing the different AI models, we earlier observed in Fig. 4 that the collective consistency, that is the one between voting outcomes corresponding to humans and those corresponding to 100% AI representation (Fig. 2), is comparable for GPT 4-o Mini and GPT3.5, with no statistically significant difference. We notice a similar trend here, where AI representation by GPT 4-o Mini achieves 2.1% higher recovery than GPT3.5, which is though, not statistically significant (p=0.092) (Figs. S5, S6). However, AI representation by GPT 4-o Mini shows significant differences in consistency recovery compared to Llama3-8B and GPT3, outperforming them by 6.4% and 8.2% respectively (Figs. S6, S7 and S8). GPT3.5 performs better than Llama3-8B and GPT3, achieving recovery gains of 4.61% and 5.97%, respectively (Figs. S6, S7, S8, and Table S11).

**AI representation of arbitrary vs. abstaining voters: from removing noise to restoring representation deficit.** Figs. 4c and 4d show the origin of inconsistency under utilitarian greedy and equal shares when voters abstain and how AI representatives recover from this. The figures show which projects are involved in consistency recovery and their ranking: (i) erroneously removed projects (false negatives, left) that are correctly added back by AI representatives (true positives) and (ii) erroneously added projects (false positives, right) that are correctly removed by AI representatives (true negatives). Compared to true negative projects, true positive ones are higher in ranking by an average of 7.2 and 2.5 positions for equal shares and utilitarian greedy, respectively. The higher consistency recovery by the abstaining models compared to the random control population originates from an average of 0.71 and 0.47 additional projects involved in consistency recovery for the two ballot aggregation methods, respectively. Moreover, the origin of consistency recovery by abstaining models is more prominent to true positive projects (mean of 1.66 over 1.0 for true negatives), while it is more prominent to true negative projects in the random control populations (mean of 2.27 over 1.89 for true positives). See Table S12 for a complete outline based on all the AI models. This result demonstrates a distinguishing quality of targeting the AI representation to abstaining voters: representation deficit is restored by adding back winners who would not be there otherwise, while a non-targeted AI representation has a noise-removal effect by removing erroneous winners. The district-wise consistency recovery for the Aarau has been enumerated in Table S13.

### Biases explaining AI (in)consistencies in choice and preference transitivity

**Unraveling biases that explain AI inconsistencies.** Fig. 5 illustrates the biases that explain the (in)consistency of human-AI choice and the AI transitivity among different ballot formats (single choice vs. cumulative). We mainly show the results of the actual participatory budgeting of City Idea, while the results of the other datasets are shown in Fig. S12. We distinguish between (i) the inconsistencies originated by the three identified subpopulations of abstained voters and (ii) the inconsistencies by the AI representation of the entire population. Prediction models are constructed using recurrent neural networks (see Sections S3.2 and S3.3), demonstrating robust performance with F1 scores averaging over 80% for abstaining groups and 74% for the entire population across all large language models (Table S16). The different personal human traits are used as features to predict the consistency between human and AI choices or between AI choices corresponding to different ballots. The relative importance of the personal human traits (independent variables) that explain the AI consistency for individual voters (dependent variable) is calculated using model agnostic shapley additive explanations and local interpretable model-agnostic explanations (see Section 4.3, Figs. S14, S15, and S16) [58]. The features that are statistically significant and have high importance scores are then analyzed to understand the types of biases based on existing literature evidence (see Section S3.1). For the abstaining models, the dependent variable is the difference (plotted in Fig. S11) between the consistency of abstaining voters and the mean of 10 random control subpopulations as shown in Section 2.2. This allows us to isolate the biases on the voters who are likely to abstain rather than on arbitrary voters. To provide more robust evidence, we distinguish in Figs. 5c and 5d those personal human traits that explain AI consistency (i) in all datasets, (ii) for GPT 4-o Mini, GPT3.5, and Llama3-8B, and (iii) those which are statistically significant (p<0.05).

**Affect and unconscious biases explain the (in)consistency of human-AI choice, while time discounting biases explain transitivity of AI choice over ballot formats.** The consistency of human-AI choice is explained by support to families (affect, 7.43%, p=0.03), public space (time discounting, 8.91%, p=0.01) and environment (conformity, 8.46%, p=0.03), while inconsistency is explained by support to elderly (affect, 11.39%, p=0.04). For the US elections, a political profile of left explains consistency of human-AI choice, while white voters explain inconsistency (33% higher than consistency, p<0.019, see Fig. S12). Affect and time discounting biases also explain the transitivity of AI representation over different ballot formats, in particular the support to families (18.22%, p=0.01), welfare (16.78%, p=0.02), and sport projects (17.05%, p=0.02). Figs. S13, S14, S15, S16, Table S18, and Section S3.4 illustrate additional insights about how personal human traits explain the AI top choice and the human-AI consistency of the individual choices for six large language models: GPT 4-o Mini, GPT3.5, GPT3, Llama3-8B, Gemini 1.5 Flash, and Deepseek R1.

## Discussion

**The inevitability of generative AI voting and the race to safeguard democracy.** Generative AI voting is likely to emerge as an inevitable technological convergence of AI and electronic voting solutions that are already being adopted in the real world. Our research does not imply or advocate the use of AI as a substitute for human voters who may choose to abstain. AI cannot replicate the human decision-making process in voting, which is shaped by socio-cultural and economic backgrounds, life experiences, and personal choices. However, generative AI and large language models are expected to become more open, pervasive, and accessible to citizens [15, 59]. AI personal assistants are already part of everyday life [60–62], with their generative version expected to follow. On the other hand, the mandate of more direct, secure, and active participation in decision-making for public matters is expected to further scale up electronic voting solutions and digital platforms. For instance, participatory budgeting elections are mainly conducted digitally, while Estonia has already institutionalized a digital identity for 99% of its citizens as well as electronic voting since 2005 [63]. As the former president of Estonia emphasized “*with the digital signature and the machine-readable ID card, we created the e-citizen*”. In the light of these converging technological advancements, the inter-operation of a generative personal voting assistant with digital voting platforms becomes technologically feasible, along with the citizens’ need to have a more direct say in several public matters and consultations. Therefore, the findings of this study become spot-on to understand the implications of such a future, while they are significant to prepare timely safeguards for digital democracy. Another inevitable risk for the integrity of elections is the use of AI representatives for running opinion and election polls at a lower cost and larger scale. This is particularly alarming given the influential role of polls to shape voting behavior and how they can often be instrumentalized to influence election results [64, 65]. Section S2.4 and Table S14 evaluate the consistency of the voting results using different sampling strategies of voters represented by AI models.

**What we can optimize for? Fair voting design as a democratic safeguard to generative AI voting.** We show that large language models currently have limitations in accurately representing individual human preferences in complex voting scenarios, such as participatory budgeting. They are also susceptible to multi-faceted biases. However, we also show that in voting scenarios involving AI representatives, voting design can play a crucial role by preserving the consistency of choices and elections as well as maximizing the recovery of consistency lost by abstaining voters. This is particularly the case for ballot aggregation methods that promote proportional representation, such as equal shares. Therefore, this motivates a huge opportunity to get democracy “right” in the digital era of AI: move to alternative voting methods that yield fairer voting outcomes for all, while shielding democratic outcomes from AI biases and inconsistencies. How to scale up these democratic blueprints remains, though, an open question. In particular, voting turnouts in participatory budgeting remain very low and far lower than in other elections, such as referenda or national elections. Despite the eminent ethical and legal challenges of engaging AI representatives in democratic processes, our findings show that a more ethically aligned AI representation of abstaining voters recovers consistency of voting outcomes, which would be lost in any case. This consistency loss can be to such a large extent that it is currently posing a long-standing barrier for participatory initiatives to take off. Note that we focus on the AI representation of abstaining voters who intend to participate but their low engagement [53, 54], digital literacy [6, 8, 12, 50, 51], or trust [52, 55, 56] are barriers for them. This is to distinguish voters whose abstention is a conscious, deliberate act, and their AI representation would not be relevant or even desired in this context. Last but not least, our findings also demonstrate that AI representation alone does not suffice - a fair voting design is imperative to materialize significant recoveries from low voting turnouts.

**Why fair collective choice is resilient to AI biases and inconsistencies.** We provide an explanation of this significant finding. There is evidence that the equal shares method has an inherent stability in the resulting voting outcomes [29]. Low- and middle-cost projects require very minimal support to get elected, and as a result, these winning projects are likely to be retained in the winning set, even with different choices or groups of voters. Such projects are expected to be a source of consistency. Indeed, this effect is also observed in the real-world voting scenario of City Idea, as with 80% abstaining voters, 84% of the winners are retained with equal shares, see Fig. S1 and Table S10 that shows the origin of this stability in terms of new projects added and removed in the winning set. Nevertheless, equal shares is still affected by low voter turnout, especially, for participation rates $<50\%$ [29]. As voter turnout in participatory budgeting is typically very low, these inconsistencies are both relevant and prevalent. Note that any comparison of stability between equal shares and utilitarian greedy should be made with caution, as the number of winning projects under equal shares is much larger than utilitarian greedy. When we control for the number of winning projects between the two methods, equal shares remains more robust than utilitarian greedy, but to a much lower extent (see baseline [random] in Figs. 4a and S6.)

**Real-world testing of equal shares: overcoming a validity barrier and addressing data limitations.** As City Idea promoted equal shares already in the project ideation phase and made use of equal shares for the aggregation of the ballots, this study becomes the first of its kind: significant findings are illustrated that come with compelling realism and merit for their validity. This comes in stark contrast to other earlier studies [28, 29] that hypothesize the application of equal shares over proposed projects and ballots aggregated with the standard method of utilitarian greedy. Access to voters’ profiles and preferences to emulate AI representation for voters is particularly limited. The City Idea participatory budgeting campaign overcame these limitations by collecting relevant data that captures such preferences to a meaningful extent. However, we also acknowledge that a broader collection of participatory budgeting elections from Pabulib [48] could not be used in our study to analyze potential biases due to the unavailability of preference data.

**Trustworthy generative AI voting: a call for research and policy action.** What information large language models use to reason about voting decisions is influential for different types of biases to manifest. This is particularly the case for affect, unconscious and time discounting biases involved in AI representation of human choices and the transitivity of AI choices over different ballot formats. Abstaining voters with low engagement, digital literacy and trust also possess related personal human traits that explain the consistency of their AI representation. Voters who come with a more active participation profile, without typical features of abstaining voters, appear irreplaceable, as the reasoning of large language models cannot accurately estimate their choices (Fig. S10). This motivates a tailored, purposeful and finite use of AI representation with the aim to make itself obsolete by recovering participation of abstaining voters, while mitigating for the consistency loss as long as voters abstain. Training data in generative AI voting are expected to play a key role for representative voting outcomes of the voter population. Ethical and democratic guidelines are urgently needed, particularly for the use of (generative) AI in voting processes. For instance, who shall determine the input training data of AI representatives? Should the training data involve only self-determined personal information of voters, or shall these be augmented with more universal knowledge and experts’ opinions? How to protect the privacy and autonomy [62] of voters when training such AI representatives? Will citizens retain power to control AI representatives that reflect their values and beliefs while remaining accountable? These are some key questions as a basis of a call for action on research and policy in an emerging era of generative AI voting.

## Methods

We show here how AI representatives are emulated and the real-world data based on which the voting scenarios are constructed. We also illustrate the evaluation approach and the studied human cognitive biases. Finally, the approach to explain the inconsistencies and biases of generative AI is outlined.

Note that p values reported for statistical significance in Section 2 (Results) are combined p values, which are based on summing log-transformed individual p values from all different runs (corresponding to different hyper-parameters) using the Fisher Method [66].

### Emulating AI representatives

The process of AI emulation using personal human traits, ballot formats, aggregation methods, and AI models is demonstrated for each of the real-world voting scenarios. Table 1 outlines the characteristics of the emulated voting scenarios.

**Table 1:** The studied dimensions across three real-world voting scenarios. They provide the necessary diversity to generalize the findings of this study as they include different number of voters, different ballot formats and aggregation methods, low and high numbers of alternatives, different personal human traits for studying a broad spectrum of biases, including both generative and predictive AI methods

Studied factors	US elections 2012, 2016, 2020	City Idea [Survey]	City Idea [Actual]
**Ballot input**			
**Personal human traits**			
Socio-demographics, Outcome expectations, Project preferences, Political interests	✗	✓	✓
Socio-demographics, Outcome expectations, Project preferences	✗	✓	✓
Socio-demographics, Project preferences, Political interests	✗	✓	✓
Socio-demographics, Outcome expectations, Political interests	✗	✓	✗
Socio-demographics, Political interests	✓	✓	✗
Socio-demographics, Political interests (only 1 feature)	✓	✗	✗
**Ballot formats**			
Single choice	✓	✓	✓
Approval	✗	✓	✓
Score	✗	✓	✗
Cumulative	✗	✗	✓
**Alternatives for voting**	2	5	33
**Ballot generation**			
**Generative AI**			
GPT 4-o Mini^*^	✓	✓	✓
GPT3.5	✓	✓	✓
GPT3	✗	✓	✓
Llama3-8B	✓	✓	✓
Deepseek R1^*^	✗	✓	✓
Gemini 1.5 Flash^*^	✗	✓	✓
**Predictive AI (ML)**			
Neural Networks	✓	✓	✓
**Ballot aggregation**			
Majority	✓	✓	✓
Utilitarian greedy	✗	✓	✓
Equal shares	✗	✓	✓
**Voters**	∼17,010 (across 3 years)	3314	505
**Emulated elections**	21	207	135

^*^Only for 1 combination of personal human traits - Socio-demographics, Outcome expectations, Project preferences, Political interests

**US elections.** The 2012, 2016, and 2020 survey waves of the American National Election Study (ANES) [67] are used. The dataset for three years contains 20,650 voters together with the respective voter socio-demographic characteristics for each of these three years: (i) racial/ethnic self-identification [white, black, Asian, Hispanic, or others], (ii) gender [male, female, others], (iii) age, (iv) ideology [extremely liberal, liberal, slightly liberal, moderate, slightly conservative, conservative, or extremely conservative], (v) political belief [democrat, republican, or independent], (vi) political interest [very interested, somewhat interested, not very interested, or not at all interested], (vii) church attendance [yes, no], (viii) whether the respondent reported discussing politics with family and friends [yes, no], (ix) feelings of patriotism associated with the American flag [extremely good, moderately good, a little good, neither good nor bad, a little bad, moderately bad, or extremely bad], and (x) state of residence. A total of 18 elections are emulated using two combinations of human traits and three AI models, including Llama, GPT3.5, and a predictive ML model for single choice ballots, with winners determined by majority aggregation for 2012, 2016 and 2020. Another 3 elections were emulated for GPT 4-o Mini based on single choice ballots, majority aggregation, and for one combination of human trait (see Table 1). We systematically removed responses containing missing data, resulting in a refined subset of 17,010 voters with complete responses. These voters were emulated using three large language models - GPT 4-o Mini, GPT3.5, and Llama3-8B for generating a total of 51,030 AI representatives. Additionally, we incorporated 3,640 voters from the original dataset who had provided partial responses specifically related to personal human traits. These incomplete cases were similarly emulated using GPT 4-o Mini, GPT3.5, and Llama3-8B, yielding 7,280 AI representatives. While these partially complete responses were utilized for vote aggregation, they were excluded from consistency prediction due to data limitations. The examples of the prompts used to generate the AI choices can be found in Table S9.

**City Idea: Participatory budgeting campaign in Aarau, Switzerland.** The data from a recent innovative participatory budgeting campaign are used [28, 36], which was conducted with ethical approval from University of Fribourg (#2021-680). It had run in 2023 and is rigorously designed to assess the application of equal shares for the first time in the real world, in combination with cumulative voting, using the open-source Stanford Participatory Budgeting platform [37]. The campaign was structured into six phases over a period of nine months. As part of this process, both pre-voting and post-voting surveys were conducted to capture the personal traits of the participant as well as their perspectives before and after the voting phase. The pre-voting survey was disseminated through physical invitation letters sent by the city council to all citizens, yielding 3,592 respondents. Of these, 808 individuals voluntarily participated in the post-voting survey. In total, 1,703 citizens participated in the voting process, of whom 252 also completed both the pre-voting and post-voting surveys. The participation achieved a gender balance, proportional representation of citizens and non-citizens, and equitable representation across the 18 districts of Aarau. As such, the field study includes a survey conducted before voting, linking the choices of survey respondents and voters. We use the following personal human traits from the survey (more information in Tables S3 and S4): (i) 9 key socio-demographic characteristics (e.g., age, citizenship, education) and 2 political interests (political beliefs and trust in democracy), (ii) preferences for 9 different types of projects and 6 beneficiaries, and (iii) 4 types of preferences for qualities of the voting outcome.

Two participatory budgeting voting scenarios are studied in the context of the real-world campaign of City Idea. The examples of the prompts used to generate the AI choices in both survey and actual voting can be found in Table S8, with more details in Section S1.

- *Survey Voting*: *Survey Voting*: Five hypothetically costed projects belonging to different categories are put for choice as part of the initial survey. Table S1 illustrates the project alternatives and their cost. The choice of 3,314 voters over the same alternatives is tested with three different ballot formats in a sequence, starting with the simplest one of single choice, to the most complex ones of approvals and score voting. The set of 3,314 voters also provided their personal trait information in the survey. This allows us to emulate 180 elections = 3 ballot formats x 4 AI models x 5 combinations of personal traits x 3 ballot aggregation methods. An additional 27 elections were emulated using all human traits, the three ballot format - (single choice, approval and score) and majority, utilitarian greedy and equal shares ballot aggregation for the GPT 4-o Mini, Deepseek R1 and Gemini 1.5 Flash. Hence a total of 207 elections have been emulated. Based on the various combinations of personal traits, we then emulated a total of 19,884 corresponding AI representatives. This included 3,314 representatives for each of the six large language models: GPT 4-o Mini, GPT3.5, GPT3, Deepseek R1, Gemini 1.5 Flash and Llama3-8B. The AI representatives have then been used to emulate elections based on the combinations of ballot format and ballot aggregation methods.
- *Actual Voting*: Using the Stanford Participatory Budgeting platform [37], 1,703 voters cast their vote using cumulative ballots by distributing 10 points to at least 3 projects of their preference, out of 33 projects in total (see Table S2 for project descriptions). A subset of 505 of these voters, which participated in the initial survey and provided their personal human traits, are used to construct the AI representatives. The ballot formats of single choice and approvals are derived from the cumulative ballots by taking the project with the most points and the projects that received any point respectively. This allows us to emulate 108 elections = 3 ballot formats x 4 AI models x 3 combinations of personal traits x 3 ballot aggregation methods. An additional 27 elections were emulated using all human traits, the three ballot formats (single choice, approval, and score), and majority, utilitarian greedy, and equal shares ballot aggregation for the GPT 4-o Mini, Deepseek R1, and Gemini 1.5 Flash. Hence, a total of 135 elections have been emulated. Of the 1,703 voters, 505 also completed the voting surveys, providing personal trait information for AI emulation. Using various combinations of these traits, we generated 3,030 AI representatives, comprising 505 representatives for each of six large language models: GPT 4-o Mini, GPT3.5, GPT3, Deepseek R1, Gemini 1.5 Flash and Llama3-8B.

**Data collection infrastructure.** Generative AI choices were collected through API prompts to large language models over two periods: from June 16, 2023, to November 8, 2023, and from April 1, 2025, to August 31, 2025. We prompted the large language models using the zero-shot learning feature [41], which does not require any specific fine-tuning. We use chain of thought prompting [68] along with context-based prompting [69] to provide a comprehensive and systematic flow of information for better interpretability. A detailed explanation for the prompt designing is provided in Section S1.2.

### Evaluation of choices by AI representatives

The emulated elections with AI representatives are compared to the real-world elections of human voters at two levels: (i) *individual choice*, i.e. the ballots, and (ii) *collective choice*, i.e. the resulting voting outcomes. Consistency is the key assessment measure, derived from the *accuracy* of individual and collective AI choices compared to human decisions and the *transitivity* across different ballot formats (see Fig. 1a).

**Consistency of individual choice.** Single choice ballots for both AI and human voters are represented as binary sequences, where a value of *1* indicates approval of a specific project, and *0* denotes disapproval of all remaining alternatives. In approval voting, each alternative is assigned either *1* (approved) or *0* (not approved). In contrast, in score voting and cumulative voting each alternative receives a score or a number of distributed points (integer numbers) reflecting voter preference. To compare AI-generated and human choices, we employ a single method, the Condorcet pairwise comparison method [13, 22], which is a generic approach to characterize the overall similarity of two ballots (or voting outcomes). A preference matrix is constructed, where rows and columns correspond to alternatives, and each matrix element records the outcome of a pairwise comparison. If project $P_{i}$ is ranked higher than project $P_{j}$, or if $P_{i}$ is approved while $P_{j}$ is not, the corresponding matrix cell $P_{i} > P_{j}$ is assigned a value of 1; otherwise, it is set to 0. Ties are excluded from the analysis.

- *Human-AI consistency (accuracy) of individual choices*: The human ballots serve as the reference point for evaluating the ones generated by the AI representatives, see Fig. 1a. The elements of ‘1’ in the matrix of AI representatives that match the elements of ‘1’ in the matrix of human choices determine the consistency [13].
- *Consistency (transitivity) of AI and human individual choice across ballot formats*: Ballot formats are standardized as follows: For cumulative/score vs. single choice ballots, the highest-scoring projects are set to ‘1’ and the others to ‘0’. For cumulative/score vs. approval ballots, scored projects are set to ‘1’, while projects without score are set to ‘0’. The elements of ‘1’ and ‘0’ in the two matrices of the ballot formats that match determine the consistency.

We also compare the choices based on preference reordering using the Kemeny distance [70] as illustrated in Section S2.

**Consistency of collective choice.** This follows the same approach of Condorcet pairwise comparisons for individual choices. However, before the calculations of consistency are made, voting outcomes are turned into binary sequences to distinguish winners (‘1’) from losers (‘0’) as determined by a ballot aggregation method.

**Consistency recovery in collective choice with AI representatives.** It is determined here for voting scenarios with varying voters turnout, in which abstained voters result in collective consistency loss, which can be recovered if a portion of these abstained voters are represented by AI. This recovery takes place at two levels: (i) False negative projects that are erroneously removed under abstaining but added back by AI representatives. (ii) False positive projects that are erroneously added under abstaining but correctly removed by AI representatives. Consistency recovery is measured as follows:

,

where the voters turnout $\frac{\text{human\ voters - abstained\ voters}}{\text{human\ voters + abstained\ voters}}$ varies in the range [20%,75%] with a step of 25%, and AI representation $\frac{\text{AI\ representatives}}{\text{abstained\ voters}}$ varies in the range [25%,100%] with a step of 25%.

### Explainability of generative AI voting

The accuracy of the individual AI choices (see Sect. S3.4) with human choices, as well as the transitivity of AI choices over different ballot formats, are modeled as the dependent variable in a predictive machine learning framework. We study causal relationships explaining how personal human traits (independent variables) influence consistency (both accuracy and transitivity). We model the problem of explaining inconsistencies as a classification problem, where 10 uniform consistency levels are defined as the ranges $[0.0,0.1],(0.1,0.2],\ldots,(0.9,1.0]$. Further details about how we account for imbalances of features, their colinearity and hyperparameter optimization of the model are illustrated in Section S3.3 and Table S15.

**Explainability of choices.** We introduce a two-dimensional feature importance analysis framework to determine the impact of the personal human traits on the consistency of individual choices. For a given performance of the prediction model, we employ explainable AI methods to analyze the contribution of each individual human trait (feature) to the outcome. The approach to enhance the performance (accuracy, precision, recall) of the prediction model is illustrated in Section S3.3. We then use the model agnostic Shapley Additive Explanations (SHAP) and Local Interpretable Model Agnostic Explanations (LIME) [58] to extract the individual contributions of each trait. Results are shown in Figs. S12, S13, S14, S15, S16 and Tables S15, S16, S17, S18. A feature ablation study [71] is used to calculate the error (loss) in the overall prediction accuracy of the model when a feature is removed (results in Table S17).

## Supplementary Information

Below is the link to the electronic supplementary material.

(PDF 8.0 MB)

## Data Availability

https://figshare.com/collections/Generative_AI_Voting_-_ANES/7261288
