# Supplementary material for: Generative AI voting: fair collective choice is resilient to LLM biases and inconsistencies
Source: EPJ Data Sci. 2026 Feb 9;15(1):24. doi: 10.1140/epjds/s13688-025-00612-3 (PMC12963128; doi:10.1140/epjds/s13688-025-00612-3)
Supplement: Supplementary file 1 — (PDF 8.0 MB) [file 13688_2025_612_MOESM1_ESM.pdf]

Generative AI voting: fair collective choice is resilient to LLM biases and inconsistencies
Supplementary Information

Srijoni Majumdar[1], Edith Elkind[2], and Evangelos Pournaras[1]

[1]School of Computer Science, University of Leeds, Leeds, UK,
E-mails: {S.Majumdar,E.Pournaras}@leeds.ac.uk
[2]Department of Computer Science, Northwestern University, Evanston, US
E-mails: edith.elkind@northwestern.edu

Contents

S1 Field study for multi-winner voting **1**
S1.1 Pre-voting and Post-voting surveys . . . 2
S1.2 Prompt design for AI representation . . . 2

S2 Consistency of AI choices **7**
S2.1 Individual and collective consistency . . . 8
S2.2 Consistency across LLMs . . . 9
S2.3 Abstaining models . . . 11
S2.4 Consistency recovery using AI representation . . . 11

S3 The machine learning framework **14**
S3.1 Human cognitive biases in AI collective decision making . . . 15
S3.2 Fairness in machine learning architectures . . . 18
S3.3 Incremental prediction of AI choice consistency based on personal human traits groups . . . 20
S3.4 Explainability of choices . . . 22

S1 Field study for multi-winner voting

This section outlines the details of the pre-voting and post-voting surveys from the 2023 participatory budgeting campaign of City Idea in Aarau. We also elaborate on the prompt design that has been used to emulate an AI representation of voters using the data collected from the surveys.

[1]Corresponding author: Srijoni Majumdar, School of Computer Science, University of Leeds, Leeds, UK, E-mail: s.majumdar@leeds.ac.uk

S1.1 Pre-voting and Post-voting surveys

The voting scenarios including the projects (alternatives) put up for voting and their characteristics, are presented in Tables S1 and S2. The personal human traits collected from the pre-voting and post-voting surveys are provided in Tables S3–S7.

S1.2 Prompt design for AI representation

We highlight the prompt design techniques, along with the approaches employed to mitigate biases introduced by the prompt specifications. Examples of prompts used to generate AI voting personas and their choices are shown in Table S8 (survey and actual voting of the City Idea participatory campaign) and Table S9 (American National Election Studies).

Prompt Design. We have designed the prompts using context based prompting [34] with the details of the voting scenarios as the voting context. The voting context primarily includes project descriptions, detailing the type of project, its location, and its impact on citizens, in addition to the ballot formats. The project descriptions are clear and unambiguous. We have further incorporated chain of thought prompting [7], where individual voter information is provided so that the language model can apply *common sense* reasoning considering the global voting context and the individual information. In addition, these models have leveraged high dimensional word embeddings [27] to effectively analyze semantic similarities between terms such as "trash cans" and bins." We run the models with temperature settings from 0.4 to 0, performing 20 runs for each setting. We calculate the consistency at each temperature setting and take the mean across all runs [34, 7]. As we are dealing with a significantly large decision space, particularly the 33 projects in the actual voting, running with very high temperature settings can lead to randomness in the generation of choices [7]. Hence we limit the range of the temperature setting from 0.4 to 0 [34, 7].

Prompt induced bias. We employ the following techniques [26] to detect and mitigate knowledge, position, and format biases, which are commonly observed in large language model generation and reasoning [8, 36].

- *Knowledge biases [8]*: To analyze and mitigate this bias, we design multiple runs in which we vary (a) the individual voter information, using different combinations of personal traits related to project preferences, voting outcome expectations, socio-demographics, and political interests, and (b) the voting context by providing projects with and without detailed descriptions. We observe that, on average, large language models generate ballots with 3 more projects in the actual voting scenario when all personal traits for individual voter information and project descriptions in the voting context are considered. This indicates that greater knowledge support helps the models generate less sparse ballots, facilitating more legitimate decision making.
- *Format biases [8]*: We experimented by providing the projects and descriptions in both tabular and list formats in the prompt, but the ballots generated did not differ in most cases. However, we observed that in 2% of ballots generated by `GPT3.5` and 4.13% of ballots generated by `GPT-4 Mini`, the tabular format produced one less project on average for the actual City Idea voting scenario. Even though this change occurred in a very small subset of the generated ballots, we still proceeded with the list format to mitigate such scenarios.
- *Position biases [36, 33]*: We tested different project orderings (ascending and descending) based on project ID and cost, as well as the original order used to present the projects for voting. The original order was not sorted by project ID or cost and was mostly based on the sequence in which the projects were proposed and registered. In most cases, the project selections in the generated ballots remained unaffected. However, for `Llama3-8B`, presenting projects in the original order resulted in ballot generation with 2 more projects on average compared to other order configurations. Therefore, we adopted the original order to include the projects in the prompt context.

Table S1: **Participatory budgeting campaign - City Idea [Survey] in Aarau**. A total of 5 projects were proposed for the survey voting which were related to urban greenery, public space, public transit and health. The total budget was set to 50,000 CHF.

ID	Project Descriptions	Cost (in CHF)
P1	Bins placed in local woodland to reduce litter	5000
P2	Recreational activities for elderly	10,000
P3	Refurbishment of local park	30,000
P4	Mental health counseling at local school	15,000
P5	Bike lane improvements	40,000

Table S2: **Participatory budgeting campaign - City Idea [Actual] in Aarau**. Citizens proposed more than 161 project ideas out of which 33 projects are selected to put for voting [29]. The proposed projects were related to education, culture, environment, welfare, urban greenery, public space, public transit, and health. The total budget was set to 50,000 CHF.

ID	Project Descriptions	Cost (in CHF)
P1	Upgrade Ruchlig soccer field	15,000
P2	Boule for all in Telli	2800
P3	Intergenerational project	1600
P4	Wild bees' paradise	20,000
P5	Parent-Child Fun and Action Day	3100
P6	Gruezi 2024 - New Year's Party	4000
P7	Children's Disco	4330
P8	Long Table Festival	3400
P9	Let's Play Football	2300
P10	LGBTQIA+ monthly party	20,000
P11	Open sports hall	2300
P12	Open closet	7000
P13	Open children's studio	10,000
P14	Petanque court	8000
P15	Pfasyl Aargau	3600
P16	Sponsoring a space for Aarau	1000
P17	Seniors gathering 70+	3500
P18	Processing birth	5000
P19	Ways of remembering	500
P20	Bread tour	1500
P21	Public bicycle pumps	4000
P22	CufA - Cultural Festival Aarau	15,000
P23	One Place for all	17,000
P24	Public herb garden	800
P25	Aarau Future Acre	3600
P26	Summer fun in the Sonnmatt summer garden	1500
P27	New edition of the Telli Map	4000
P28	Climate days for Aarau	24,000
P29	A Garden for All	2500
P30	Summery cinema nights in the Badi	10,000
P31	Ruchlig water playground	25,000
P32	Usable space with a hedge	1000
P33	Playground extension Oehlerpark	20,000

Table S3: Pre-voting (Pr) survey : Socio-demographics, political interests and outcome expectations

ID	Question	Type	Options
	**Socio-demographic characteristics**		
SPr.1	What is your gender?	Single Choice	3 [man, woman, various/ other]
SPr.2	What is your age?	Number	String
SPr.3	What is your location?	Text	String
SPr.4	Are you entitled to vote in Switzerland?	Single Choice	2 [yes, no]
SPr.5	What is the highest education you have completed so far?	Single Choice	5 [school level, bachelors, masters, doctorate and above]
SPr.6	Were you born in Switzerland?	Single Choice	4 [no, yes, don't know, no answer]
SPr.7	Did your parents migrate to Switzerland?	Single Choice	5 [yes both, only one, no both parents immigrated, don't know, no answer]
SPr.8	Do you have children?	Single Choice	3 [no, yes, no answer]
SPr.9	Do you have trust in political parties	Single Choice	3 [no, yes, no answer]
	**Political interests**		
IPr.1	Where would you place yourself on a scale from 0 to 10, on which 0 means "left" and 10 means "right"?	Ratio Scale	12 [extremely left to extremely right, don't know, no answer]
IPr.2	How interested are you in politics in general?	Ratio Scale	6 [not interested at all, rather not interested, somewhat interested, very interested, don't know, no answer]
IPr.3	On a scale from 0 (no trust) to 10 (full trust), how much do you trust the following institutions, organizations and groups?	Group	2 questions
IPr.3.1	City council (government)	Ratio Scale	10 [no trust, very low trust, low trust, moderate trust, neutral, moderate trust, moderate high trust, high trust, very high trust, full trust]
IPr.3.2	Social media	Ratio Scale	10 [no trust, very low trust, low trust, moderate trust, neutral, moderate trust, moderate high trust, high trust, very high trust, full trust]
	**Outcome expectations**		
VPr.1	Which method to you prefer for the selection of the projects? Please rank them from 1 to 3. Options are Method 1: most votes, Method 2: most of the budget, Method 3: satisfy most voters	Multiple - Ratio Scale	5 [most preferred, second most preferred, third most preferred, don't know, no answer]
VPr.2	On a scale of 1 to 5, how important do you think these criteria are for the selection of projects to implement at a local level? (such as measures for climate adaptation or economic promotion)?	Group	4 questions
VPr.2.1	Cost efficiency	Ratio Scale	7 [not important, very less important, moderately important, important, highly important, don't know, no answer]
VPr.2.2	Environmental impact	Ratio Scale	7 [not important, very less important, moderately important, important, highly important, don't know, no answer]
VPr.2.3	Benefit for city	Ratio Scale	7 [not important, very less important, moderately important, important, highly important, don't know, no answer]
VPr.2.4	Benefit for myself	Ratio Scale	7 [not important, very less important, moderately important, important, highly important, don't know, no answer]

Table S4: Pre-voting (Pr) survey : Project preferences

ID	Question	Type	Options
PPr.1	You now see nine thematic areas in which urban projects can be realized. Please select the ones you support. The nine areas are Education, Urban greenery (e.g. parks, greenery), Public space (e.g. squares), Welfare (for people living below the poverty line), Culture, Environmental protection, Public transit and roads, Sports, and Health	Multiple choice	2 [no, yes]
PPr.2	On a scale of 1 to 5, how important is it to you that the following group benefits from urban projects?	Group	6 questions
PPr.2.1	Families with children	Ratio Scale	7 [not important, very less important, moderately important, important, highly important, don't know, no answer]
PPr.2.2	Children	Ratio Scale	7 [not important, very less important, moderately important, important, highly important, don't know, no answer]
PPr.2.3	Youth	Ratio Scale	7 [not important, very less important, moderately important, important, highly important, don't know, no answer]
PPr.2.4	Adults	Ratio Scale	7 [not important, very less important, moderately important, important, highly important, don't know, no answer]
PPr.2.5	People with disabilities	Ratio Scale	7 [not important, very less important, moderately important, important, highly important, don't know, no answer]
PPr.2.6	Elderly	Ratio Scale	7 [not important, very less important, moderately important, important, highly important, don't know, no answer]

Table S5: Pre-voting (Pr) survey: Digital literacy

ID	Question	Type	Options
DPr.1	To what degree do the following statements apply to you?	Group	2 questions
DPr.1.1	I know how to adjust the privacy settings on a mobile phone or tablet	Ratio scale	7 [completely disagree, disagree, neutral, agree, completely agree, don't know, no answer]
DPr.1.2	I tend to shy away from using digital technologies where possible.	Ratio scale	7 [completely disagree, disagree, neutral, agree, completely agree, don't know, no answer]
DPr.2	In general, how much trust do you have in online voting / e-voting solutions?	Ratio scale	6 [no trust at all, rather no trust, rather trust, a lot of trust, don't know, no answer]

Table S6: Pre-voting (Pr) and Post-voting (Po) survey: Engagement profile

ID	Question	Type	Options
EPr.1	How often do you interact with the following persons?	Group	2 questions

Continued on next page

Table S6 – continued from previous page

ID	Question	Type	Options
EPr.1.1	Other inhabitants of Aarau	Ratio Scale	7 [daily, weekly, quarterly, annually, never, don't know, no answer]
EPr.1.2	Members of Residents' Council	Ratio Scale	7 [daily, weekly, quarterly, annually, never, don't know, no answer]
EPo.6	What were your reasons to participate in the Stadtidee vote? You may tick more than one answer.	Multiple choice	11 [support for one or more projects, interest in a new form of participation, civic duty, to have my say on how the local budget is spent, to know what Stadtidee is about, to experience the online voting platform, someone encouraged me, many others have also participated, other reason (please state), don't know, no answer]

Table S7: Post-voting (Po) survey: Trust

ID	Question	Type	Options
TPo.1	What's your impression of the Stadtidee voting result? Rate the following statements on a scale from 0 (do not agree at all) to 10 (fully agree).	Group of questions	4 questions
TPo.1.1	I am satisfied with the outcome	Ratio scale	13 [do not agree at all [0] to fully agree [10], don't know, no answer]
TPo.1.2	I accept the outcome	Ratio scale	13 [do not agree at all [0] to fully agree [10], don't know, no answer]
TPo.1.3	I was able to influence the outcome	Ratio scale	13 [do not agree at all [0] to fully agree [10], don't know, no answer]
TPo.1.4	I feel the outcome of the Stadtidee votes accurately represents the will of Aarau citizens	Ratio scale	13 [do not agree at all [0] to fully agree [10], don't know, no answer]

Table S8: **Prompt design to construct AI voting personas for participatory budgeting campaign - City Idea [Survey] and [Actual] in Aarau**. The prompts are shown for selected ballot formats and personal human traits, using projects from the survey voting scenario.

Personal human traits	Prompts
Socio-demographics. Approval ballot	Among the following list of projects: P1: Bins for Litter, cost is 5000 CHF; P2: Elderly Fun, cost is 10,000 CHF; P3: Local Park, cost is 30,000 CHF; P4: Mental Health, cost is 15,000 CHF; P5: Bike Lane, cost is 40,000 CHF with a total budget of 50,000 CHF<br><br>*Which projects are preferred for a person with the following profile?*<br><br>male, 49.0 years old, lives in Zelgli, citizen of Switzerland, has education at the level of Master's degree, not born in Switzerland, whose both parents were born in Switzerland, does not have children
Political interests. Score ballot	Among the following list of projects: P1: Bins for Litter, cost is 5000 CHF; P2: Elderly Fun, cost is 10,000 CHF; P3: Local Park, cost is 30,000 CHF; P4: Mental Health, cost is 15,000 CHF; P5: Bike Lane, cost is 40,000 CHF with a total budget of 50,000 CHF<br><br>*Assign a score of 1 to 5, 5 being the highest and 1 being the lowest to the projects for a person with the following profile*<br><br>has neutral political orientation (score 5), where 1 is left wing orientation and 10 is right wing orientation, not interested in local politics of Aarau, scores the trust in city administration with 4 (moderate trust) , scores the trust in social media with 3 (low trust) where 1 is no trust and 10 is full trust.
Project preferences. Single choice	Among the following list of projects: P1: Bins for Litter, cost is 5000 CHF; P2: Elderly Fun, cost is 10,000 CHF; P3: Local Park, cost is 30,000 CHF; P4: Mental Health, cost is 15,000 CHF; P5: Bike Lane, cost is 40,000 CHF with a total budget of 50,000 CHF<br><br>*Which one is the most preferred for a person with the following profile?*<br><br>considers projects related to education as not important, urban greenery as not important, public space as important, welfare as not important, culture as not important, environmental protection as important, public transit as not important, sports as important, health as not important<br><br>scores projects that impact the elderly population with 3 (moderately important), children with 4 (important), youth with 4 (important), the adults with 2 (very less important), people with disabilities with 3 (moderately important), elderly population with 3 (moderately important) where 1 is not important and 5 is highly important

Table S9: **Prompts to construct AI voting personas for American National Election Studies - 2012, 2016 and 2020.** We have used the same prompts as used in the study of Arghyle et al. [3].

Personal human traits	Prompts
Socio-Demographics	Which candidate - Barack Obama or Mitt Romney would be most preferred in the US presidential elections 2012 for a person with the following profile?<br><br>Racially the person is black. Ideologically, the person is extremely liberal. Politically, the person is a Democrat. The person attends church. The person is 86 years old. The person is a woman. The person has no interest in politics. The person feels a little good while seeing the American flag.

S2 Consistency of AI choices

Voter abstention can influence voting outcomes in collective decision-making. Our analysis reveals that when more than 50% of voters abstain, the average changes (additions and deletions) in winning projects compared to original set of winners is 2.14 for equal shares and 3.31 for the utilitarian greedy aggregation (Table S10). The projects selected as winners with equal shares are highly resilient to abstentions; even with 80% abstaining,

Table S10: **The winners elected by the equal shares method show greater resilience than utilitarian greedy in retaining projects from the original winner set corresponding to 100% turnout.** We study project changes, including additions and deletions, by emulating election instances with abstaining voters under different aggregation methods. Abstaining voters are randomly sampled from the population using sizes of 10%, 25%, 40%, 50%, 75%, and 85%, and for each size, the random sampling process is repeated 40 times.

	Considering all elections		Considering only elections where the winners change	
	Consistency loss: Avg. changes in winners (addition + deletion)			
voters (% who abstain)	equal shares	utilitarian greedy	equal shares	utilitarian greedy
10	0.71	1.32	0.33	1.62
25	1.38	2.36	0.97	2.19
40	1.66	2.81	1.77	3.12
50	1.87	3.59	2.37	3.59
75	2.27	4.52	3.56	4.52
85	2.45	4.82	3.86	4.82

around 83.1% of the winners are retained from the original project winner set corresponding to 100% turnout (refer Figure S1).

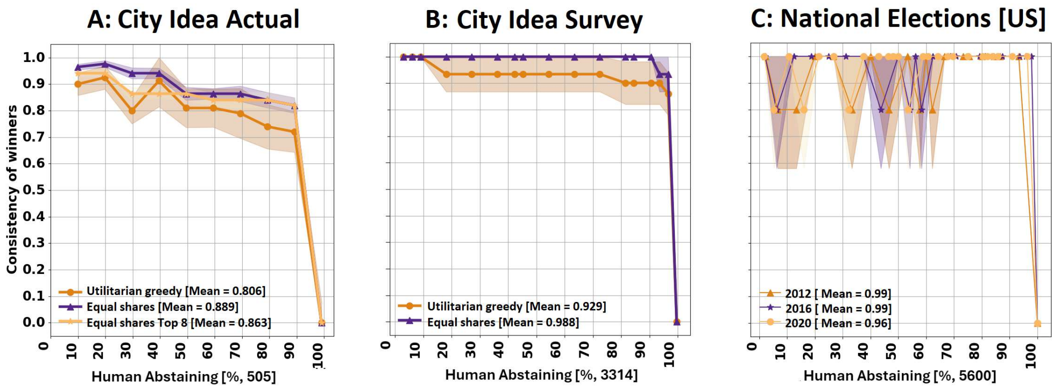

Figure S1: **Equal shares preserve 83.1% of the winners even with 80% of the voters abstaining**. The abstaining voters are randomly sampled to analyze the change in the overall decision outcomes. We use 40 iterations of random sampling and report the average consistency.

S2.1 Individual and collective consistency

The Condorcet method of pairwise comparisons [22, 28] is used to assess the accuracy of human-AI choices at both individual and collective levels. The standardization of AI and human ballots into a uniform preference matrix for project pairs is detailed in Section 4.2 (main paper). We further analyze Figure 2 (main paper) using Figure S2 to show the individual consistency representations for the different population sizes.

In addition to the Condorcet method, we also evaluate consistency using other similarity metrics, such as the Kemeny distance (Figure S3) [2]. The Kemeny distance metric measures the number of pairwise inversions needed to align the choice preference orders in two ballots. The trends in collective and individual consistency between human and AI choices using the Kemeny distance and the Condorcet methods are similar (Figures 2 (main paper) and S3).

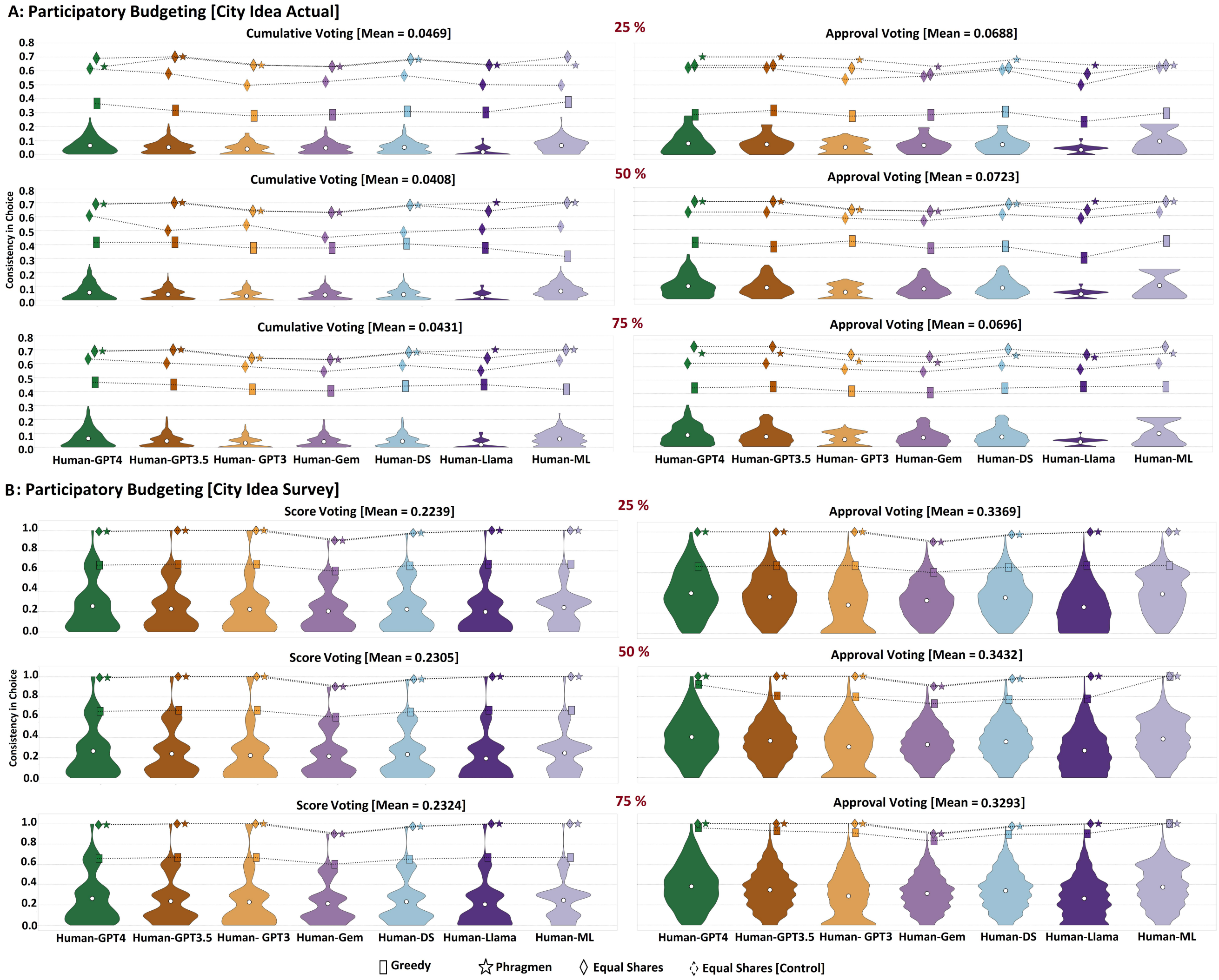

Figure S2: **The consistency of collective decision-making is significantly higher than that of individual AI choices, especially under fairer ballot aggregation rules such as equal shares and Phragmén's methods. This holds true even within voter subpopulations and when selecting from 33 voting alternatives.** The consistency (y-axis) in individual and collective choice is shown for different AI models (x-axis) for six large language models - `GPT 4-o Mini` , `GPT3.5` , `GPT3` , `Gemini 1.5 Flash` (Gem), `Deepseek R1` (DS) and `Llama3-8B` (Llama) along with the predictive AI model (*ML*), across the (A) actual and (B) survey participatory budgeting campaign of City Idea for 25%, 50% and 75% of the population. For participatory budgeting, the ballot formats of cumulative (left) and approval (right) are shown, including the ballot aggregation methods of equal shares, Phragmén's and utilitarian greedy. In case of equal shares in the actual voting, the accuracy of winners is calculated for all winners a controlled number of winners (as many as utilitarian greedy) for a fairer comparison.

S2.2 Consistency across LLMs

Human-AI Consistency: The consistency between human and AI choices are shown in Figure 2 (main paper).

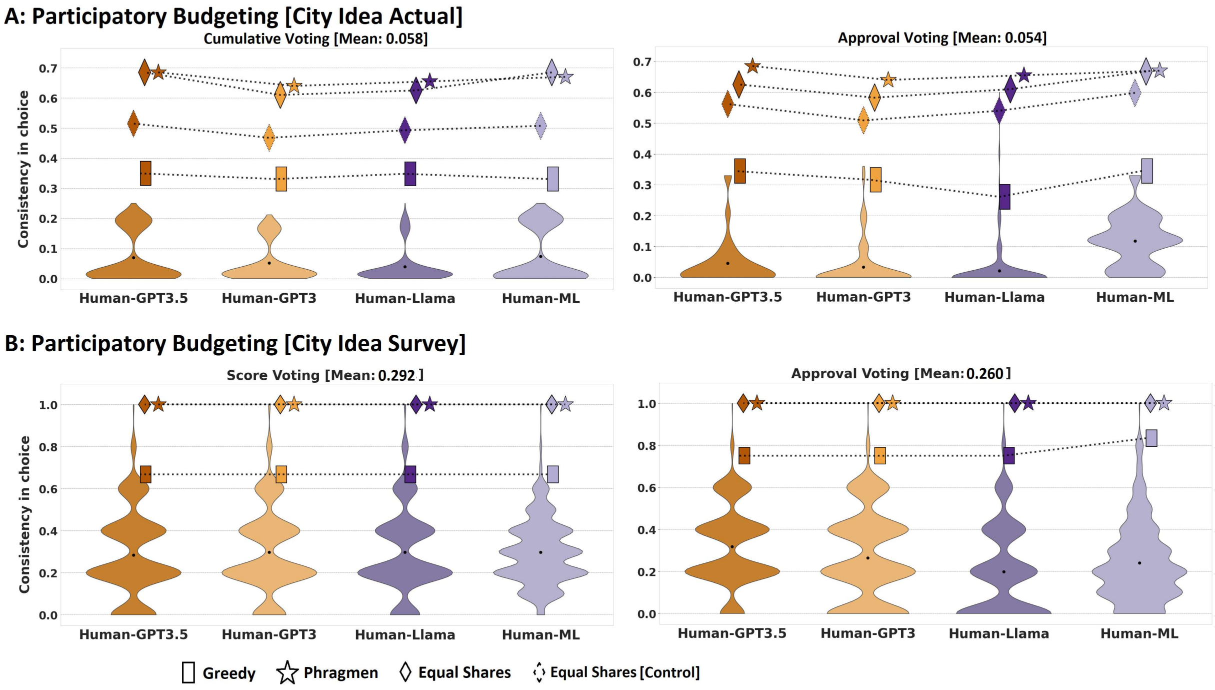

Figure S3: **The consistency of collective choice is higher than individual choice, particularly for the fairer ballot aggregation rules of equal shares and Phragmén's.** The consistency (y-axis) in individual and collective choice is shown using the Kemeny distance for different AI models (x-axis), across two real-world voting scenarios: The participatory budgeting campaign of City Idea, (A) actual and (B) survey. For participatory budgeting, the ballot formats of cumulative/score (left) and approval (right) are shown, including the ballot aggregation methods of equal shares, Phragmén's and utilitarian greedy. For the actual voting of City Idea, the consistency of equal shares is calculated for all winners and a controlled number of winners (as many as utilitarian greedy) for a fairer comparison.

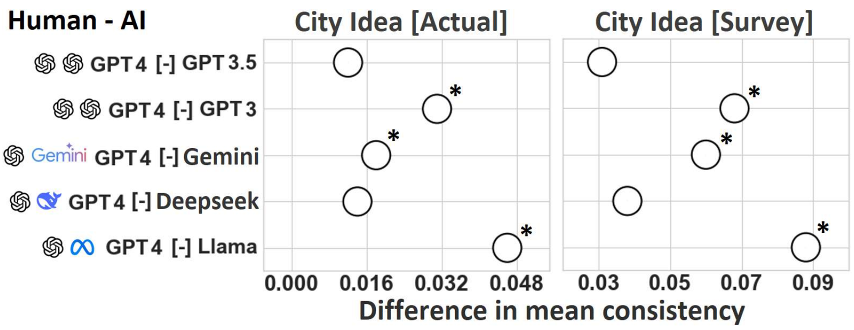

Figure S4: The difference in mean human-AI consistency (x-axis) for individual choice is shown for `GPT 4-o Mini` and five other large language models: `GPT 4-o Mini`, `GPT3.5`, `GPT3`, `Gemini 1.5 Flash` (Gemini), `Deepseek R1` (Deepseek), and `Llama3-8B` (Llama). This represents the average difference in consistency, considering probable and score/cumulative ballots. * indicates that the difference is statistically significant.

For ballots with a significant number of alternatives, `GPT 4-o Mini`, the entry level reasoning model, achieves the highest consistency, outperforming `GPT3.5` and `GPT3` by 4.7% (combined $p<0.03$) and 6.9% (combined $p<0.02$), respectively. Compared with open source models, `GPT 4-o Mini` achieves 4.72% (combined $p<0.04$) and 10.2% (combined $p<0.02$) higher consistency than `Deepseek R1` and `Llama3-8B`, respectively. In case of ballots with fewer alternatives, `Deepseek R1` shows relatively higher consistency but remains 3.9% lower than `GPT 4-o Mini`. `GPT 4-o Mini` further achieves 4.6%, 4.9%, and 8.04% higher consistency than `Gemini 1.5 Flash`, `GPT3.5`, and `GPT3`, respectively. Compared to the proprietary reasoning model `Gemini 1.5 Flash`, `GPT 4-o Mini` demonstrates 4.92% (combined $p<0.03$) and 4.69% (combined $p<0.04$) higher consistency. Overall, `GPT 4-o Mini` exhibits inconsistencies that are comparable to those of the predictive machine learning model (see Figure S4).

Consistency between AI ballots: The consistency between the different AI ballots has been demonstrated in Figure 3 (main paper). Among the open-source models, `Llama3-8B` achieves the highest consistency across different ballot formats for AI choices, with an average of 76.2%. This is followed by `GPT 4-o Mini` (74.3%), `GPT3.5` (72.1%), `Gemini 1.5 Flash` (71.23%) and `Deepseek R1` (68.7%).

S2.3 Abstaining models

We present the degree of overlap between the abstaining models. Among the 252 voters who took part in both the pre- and post-voting surveys and the actual voting, 115 have low digital literacy, 126 have low engagement interest, and 106 have low trust in institutions. 10 voters have all three traits, low digital skills, low engagement, and low trust. Additionally, 25 voters have both low trust and low engagement, 23 have low digital skills and low trust, and 28 have low digital skills and low engagement. The minimal overlap among these groups validates the approach of separate abstaining groups in the voting scenario.

S2.4 Consistency recovery using AI representation

In this section, we present additional findings on assessing consistency recovery by AI representatives, which extend the findings shown in Section 2.2 (main paper). Figures 4 (main paper) and S5 illustrate the consistency recovery using AI representation of voters who are likely to abstain, and two aggregation methods: equal shares [32] and utilitarian greedy [31], for the actual voting of City Idea, modeled using `GPT3.5` and `GPT 4-o Mini`, respectively. Additionally, Figure S6 demonstrates consistency recovery for another fair aggregation method, Phragmén's [4] method, alongside a controlled instance of equal shares, ensuring the number of winners is the same as utilitarian greedy aggregation, using `GPT3.5` and `GPT 4-o Mini`. Similarly, Figures S7 and S8 depict the consistency recovery for the actual voting scenario using utilitarian greedy, equal shares, Phragmén's, and equal shares with controlled winners for `GPT3` and `Llama3-8B`, respectively. The results on consistency recovery by AI representatives in the survey voting scenario of City Idea have been shown in Figure S9. Our findings reveal that AI representation substantially enhances consistency recovery for abstaining voter groups but has a negligible effect when applied to voters who come with a more active participation profile, and without typical features of abstaining voters (Figure S10).

Consistency recovery is at two levels: (i) False negative projects removed under abstaining but added back by AI representatives, which are are higher in ranking and number than (ii) false positive projects added under abstaining but removed by AI representatives. Detailed comparisons of false-negative and false-positive projects are provided in Figure 4 (main paper) for `GPT3.5` and in Table S11 for `Llama3-8B` and `GPT3`. The average project recovery rates for false negatives and false positives, based on abstention models and their respective random control populations, are presented in Table S12.

We further analyze the recovery of voting outcomes by examining abstention patterns across different regions (Table S13).

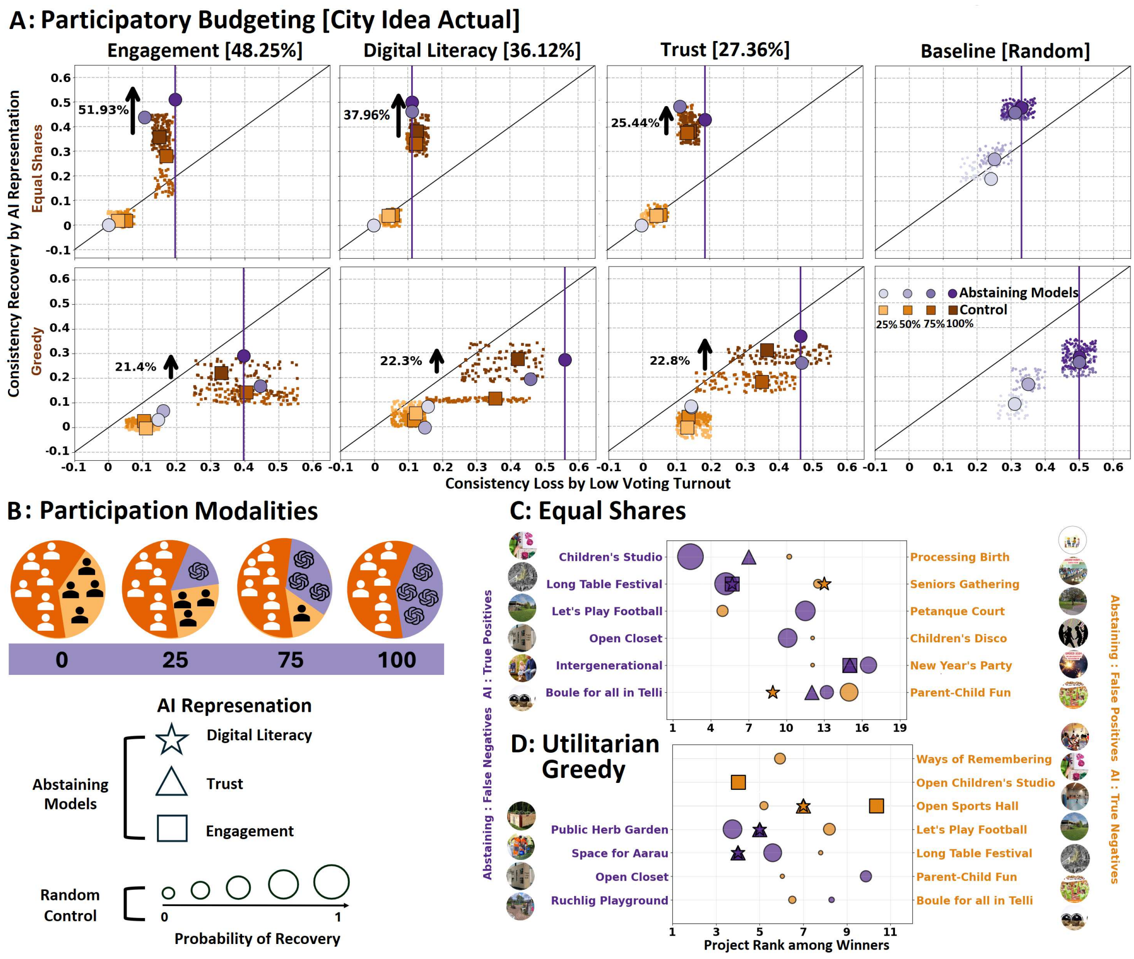

Figure S5: **Representing more than half of human abstaining voters with AI results in significant consistency recovery, in particular for fair ballot aggregation methods.** The consistency loss in voting outcomes by low voters turnout (x-axis) is emulated by removing different ratios of human voters (25%, 50%, 75% and 100%) among the whole population (baseline) and those who are likely to abstain: low engagement, trust and digital literacy profile (% of the abstaining populations in the brackets on top). A consistency recovery (y-axis) is hypothesized by AI representation using `GPT 4-o Mini` for the (A) actual participatory budgeting campaign of City Idea, (B) studied participation modalities, (C)-(D) origin of consistency recovery in participatory budgeting for utilitarian greedy and equal shares respectively. Abstaining voters result in falsely removing (left) and erroneously adding (right) winning projects. AI representatives add back and remove these projects respectively to recover consistency. The projects and their probability to recover consistency under random control are shown for comparison.

We also compare how the pre-election predictions fare against actual polls with human voters and 100% AI representation for the US national elections of 2012, 2016, and 2020. Interestingly, for the partisan dataset, the predicted winners in the pre-election closely match the actual election winners (for both humans and AI representation), except in 2016, where the pre-election prediction differed. We have taken a subset of the actual election votes and show the relative percentage of votes each candidate received in Table S14.

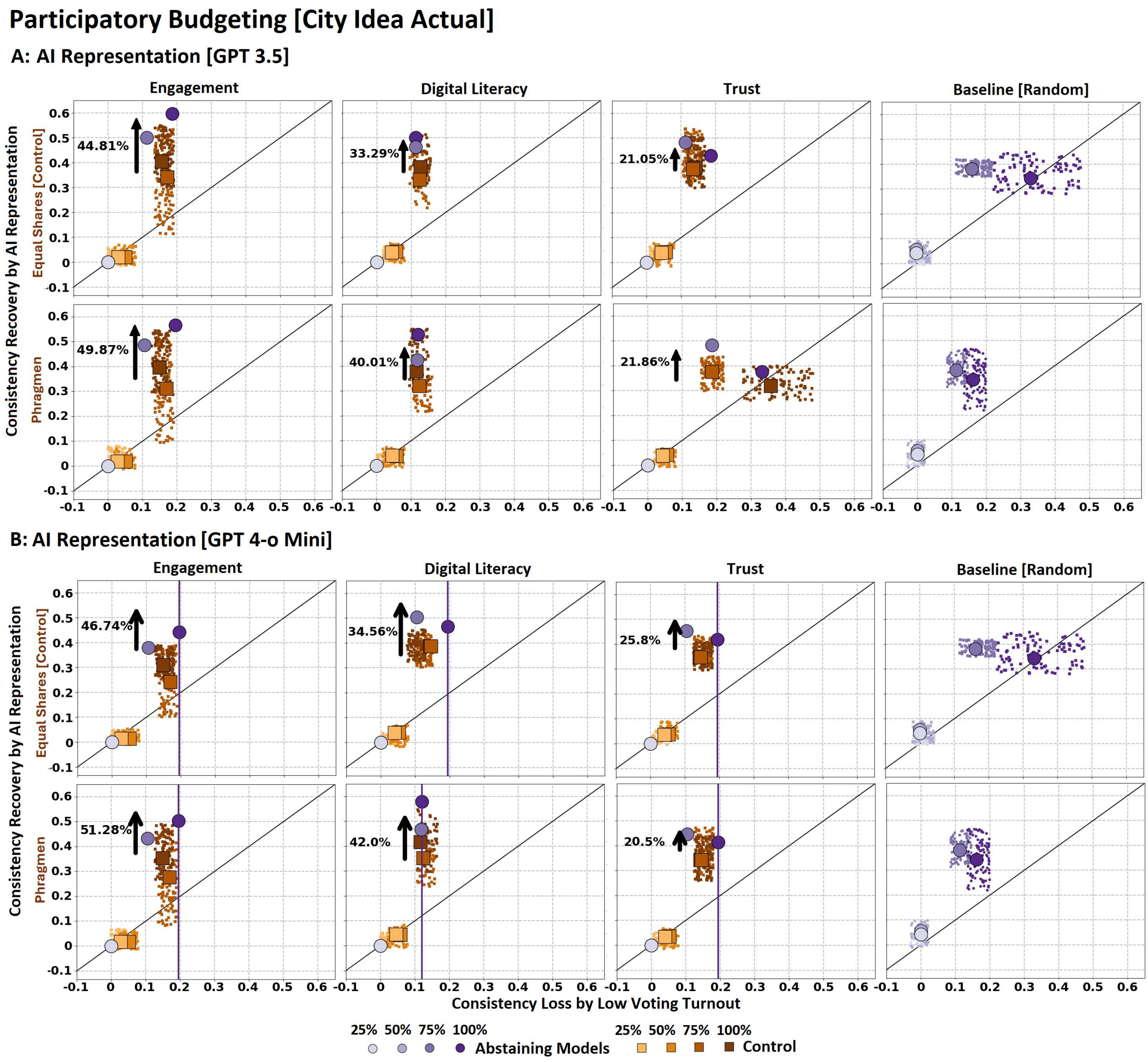

Figure S6: **AI representation of abstaining voters is more effective than representing arbitrary voters (random control) under the fair aggregation rules of Phragmén's method and equal shares (controlled settings with number of winners same as utilitarian greedy).** The consistency loss in voting outcomes by low voters turnout (x-axis) is emulated by removing different ratios of human voters (25%, 50%, 75% and 100%) among the whole population (baseline) and those who are likely to abstain: low engagement, trust and digital literacy profile. A consistency recovery (y-axis) is hypothesized by AI representation using (A) `GPT3.5` and (B) `GPT 4-o Mini` for the actual participatory budgeting campaign of City Idea.

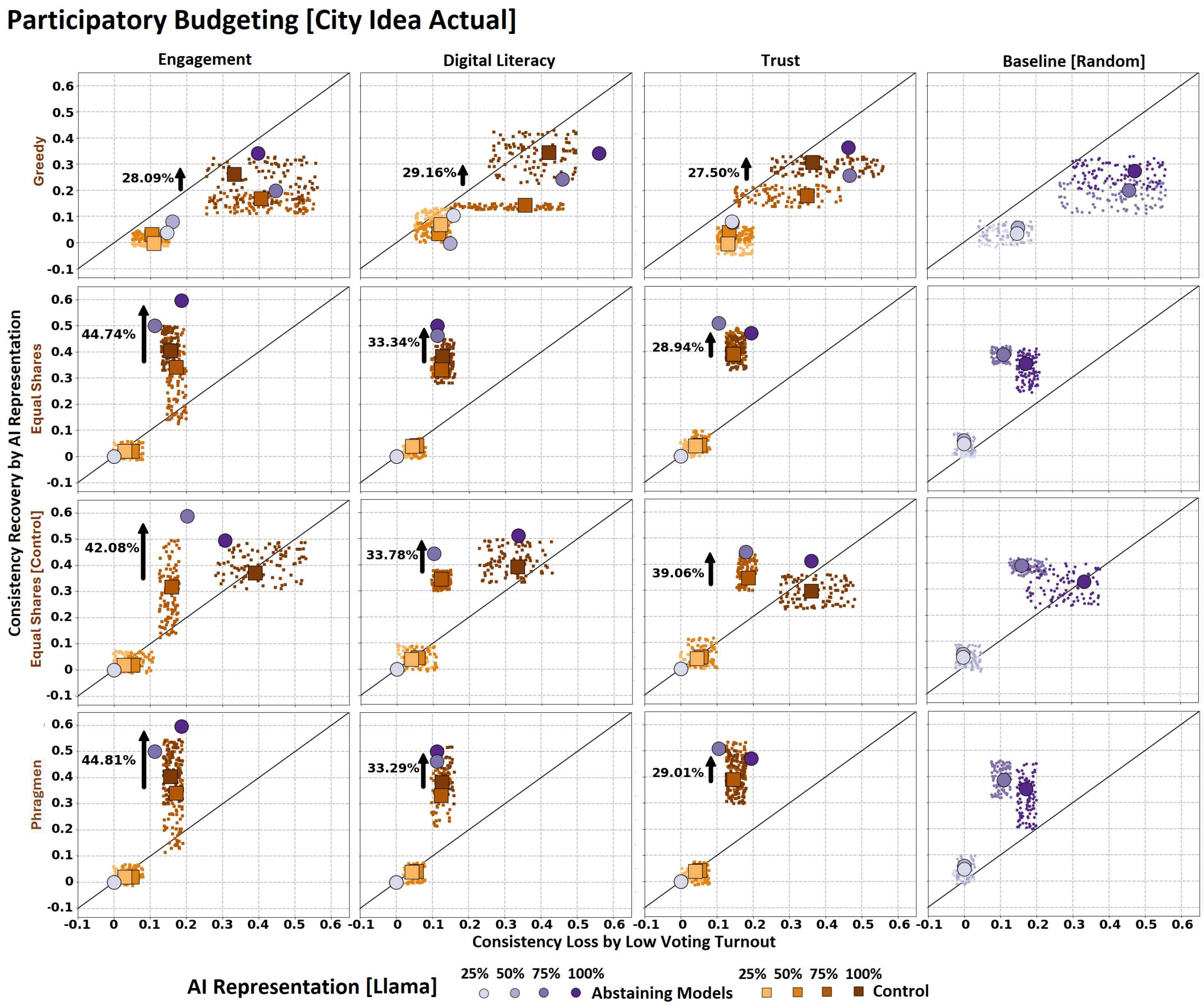

Figure S7: **AI representation of abstained voters is more effective than representing arbitrary voters (random control).** The consistency loss in voting outcomes by low voters turnout (x-axis) is emulated by removing different ratios of human voters (25%, 50%, 75% and 100%) among the whole population (baseline) and those who are likely to abstain: low engagement, trust and digital literacy profile. A consistency recovery (y-axis) is hypothesized by AI representation using `Llama3-8B` for the actual participatory budgeting campaign of City Idea.

S3 The machine learning framework

We discuss the machine learning architecture for predicting consistency gain or loss for individual voters based on their personal human traits in this section. The relevant personal human traits are mapped to cognitive biases for further analysis.

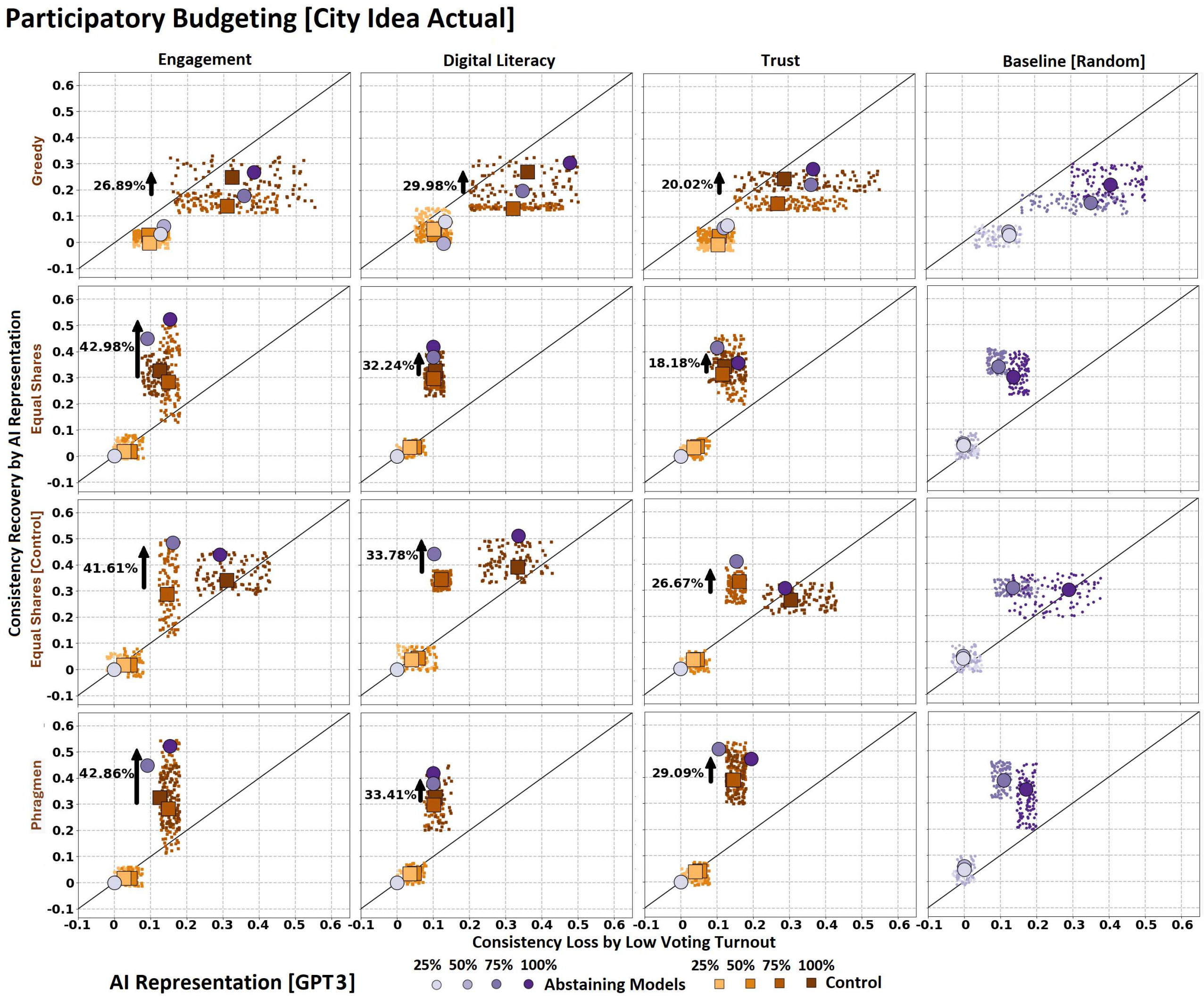

Figure S8: **AI representation of abstained voters is moderately effective than representing arbitrary voters (random control).** The consistency loss in voting outcomes by low voters turnout (x-axis) is emulated by removing different ratios of human voters (25%, 50%, 75% and 100%) among the whole population (baseline) and those who are likely to abstain: low engagement, trust and digital literacy profile. A consistency recovery (y-axis) is hypothesized by AI representation using `GPT3` for the actual participatory budgeting campaign of City Idea.

S3.1 Human cognitive biases in AI collective decision making

Human choices are significantly influenced by potential cognitive biases that are often a manifestation of socio-economic characteristics, conditions of life quality, (dis)satisfaction with the available public amenities and the overall life experiences of an individual [20]. We map self-reported personal traits to potential underlying human cognitive biases. These traits are part of the input context for ballot generation in large language models. Our goal is to explore whether these biases are reinforced by the models. If so, they may become

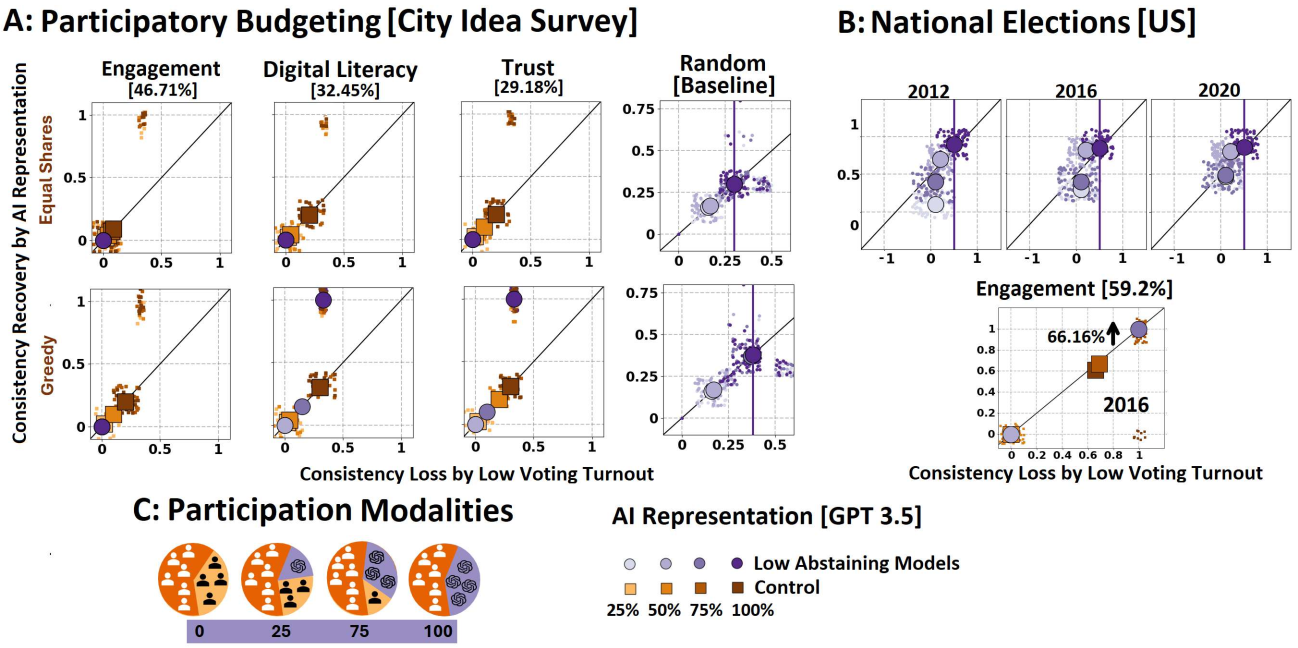

Figure S9: **Representing more than half of human abstaining voters with AI results in significant recovery of consistency, in particular for fair ballot aggregation methods such as equal shares in participatory budgeting. Strikingly, for voters likely to abstain, collective consistency would remain intact when using equal shares without any AI representation. However, the consistency loss under the utilitarian greedy approach is recovered through AI representation, proving more effective than representing an equivalent number of random voters.** The consistency loss in the voting outcome by low voters turnout (x-axis) is emulated by removing different ratios of human voters (25%, 50%, 75% and 100%), who are likely to abstain with a low engagement, trust and digital literacy profile. A recovery of consistency (y-axis) is hypothesized by AI representation using `GPT3.5`. The (A) survey voting in the participatory budgeting campaign of City Idea, (B) US elections and (C) the studied participation modalities.

more likely to manifest under AI representation. Figure 1d (main paper) outlines the mapping we study based on a systematic and comprehensive review of relevant literature. The following types of biases are determined:

Time-discounting biases. These are characterized by the tendency to receive immediate gratification over a larger but future reward. Projects related to public spaces or culture often focus on events such as annual festivals or cinema nights (alternatives proposed in the participatory budgeting campaign in Aarau [29]). These projects have a quick turnaround time, offer direct and tangible rewards, and may also create long-term, repeatable impacts. Similarly, the welfare projects proposed in Aarau [29] involve small-scale initiatives such as educating asylum-seeking children, commemorative activities, and bread tours for the elderly, all of which yield rapid benefits. Therefore, such project are subject of time-discounting biases [20].

Optimism bias. Projects such as road construction require significant time and investment costs for resources even before implementation begins. Additionally, uncertainties and challenges related to costs, infrastructure, and planning may arise during execution and delay the project from materialising. Despite these hurdles, such investments contribute to sustainable reforms that benefit society in the long run, fostering optimism among

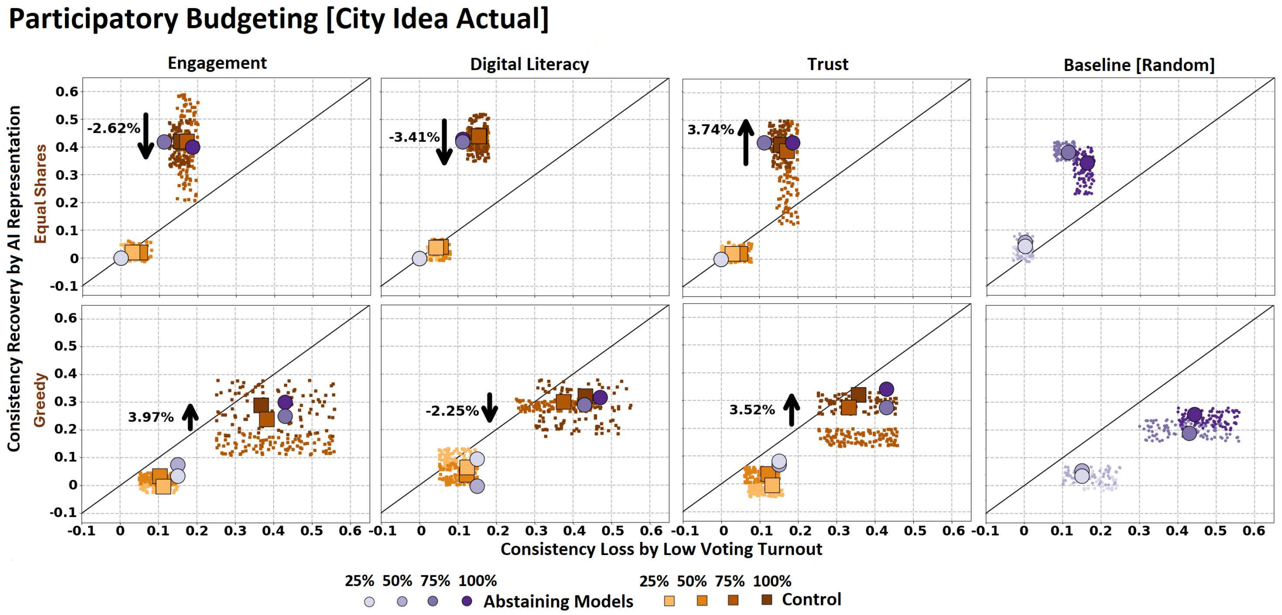

Figure S10: **The AI representation of voters who come with a more active participation profile, without typical features of abstaining voters, is not significantly more effective than representing arbitrary voters (random control).** The consistency loss in voting outcomes by low voters turnout (x-axis) is emulated by removing different ratios of human voters (25%, 50%, 75% and 100%) among the whole population (baseline) and those who are likely to abstain: low engagement, trust and digital literacy profile. A consistency recovery (y-axis) is hypothesized by AI representation using `GPT3.5` for the actual participatory budgeting campaign of City Idea.

people who continue to support them. Even though 72% of public transportation infrastructure projects in European cities experience cost overruns, voters still back these initiatives due to an inherent optimism about improving transportation [20, 23]. We refer to this tendency to prioritize long-term sustainability despite economic uncertainties as ‘optimism’ [17, 30].

Surrogation biases. This reflects how humans favor simpler measures to assess the impact over ones that are more precise and harder to evaluate. Korteling et al. [20] argue that these biases manifest when deciding projects with large societal impact such as health or education, while their outcome is subject of different satisfaction levels among citizens. The project outcomes may be perceived as successful by part of society using easy-to-evaluate metrics instead of looking at long-term effects on the community. For instance, a timely vaccination drive may be preferred over significant changes to vaccination protocols or health insurance policies covering vaccination. Hence these projects are likely to be more preferred as they come with more intuitive ways to assess for the broader population. This is reflected by the average winning rate of 38.1% and 36.2% for health and education-related projects in Poland [23], where participatory campaigns have been actively hosted in the last decade.

Conformity biases. These biases arise out of group pressure under which people make decisions to be socially desirable [10, 11]. It is argued that a conformity bias may induce voting for green alternatives [20]. Green-themed participatory budgeting campaigns have been adopted in European cities such as Lisbon [13], to promote green initiatives, aligning to a culture for more sustainable behavior. Poland runs participatory budgeting campaigns at large scale, which include environment and urban greenery projects. These are within

the top-5 most popular projects with an average of 22.5% and 26.5% respectively [23]. Even in Aarau we observe the same trend wherein, with environmental friendly projects accounting for the top-10 most popular projects [29].

Affect heuristic biases. This is the tendency to make decisions based on what intuitively or emotionally feels right. Affect biases have been studied to analyze the inclusive attitudes most people show towards elderly people [15]. Similar biases also manifest in welfare of children and in inter-generational communication [20, 1]. In Aarau, we observe that 71.3% of the voters prefer projects for younger and elderly people.

Biases for altruism and egotism. Individual interest is often in conflict with the community interest in participatory and collective decision-making processes. Intrinsic altruism of citizens influences voting choices. As a result, altruism and egotism are influential for the fairness of voting outcomes and how these outcomes benefit the city in overall [14, 20, 1]. In Aarau, we observe that 67.1% of the voters, who prefer better representation in the outcome are prosocial and prioritize city-wide benefit (altruism bias) over individual benefit (egotism bias).

Unconscious biases. Human choices are influenced by socio-economic and demographic traits such as race, ethnicity, citizenship, household size and income [9]. Specifically, political ideology and belief shape to a high degree decisions for candidates in elections [21].

Table S11: **Voter abstention can cause incorrect removal of projects (false negatives), or incorrect addition of projects (false positives), in the winning set compared to the original winner set at 100% turnout.** The findings are shown for the AI representation using `Llama3-8B` and `GPT3`.

**Project types**<br>**Aggregation, AI models**	**Projects**	**Probability**	**Rank**	**Abstaining models, Rank**
False negatives	Boule for all in Telli	0.34	14	Digital literacy, 13
Equal shares, `Llama3-8B`	New edition of Telli map	0.42	12.5	Digital literacy, 13; Engagement, 12
	Open Sports Hall	0.83	10.7	
	Long Table Festival	0.81	5.4	Digital literacy, 13; Engagement, 12; Trust, 13
	Let's Play Football	0.65	9.5	
False positives	A Garden for all	0.49	12.6	
Equal shares, `Llama3-8B`	Petanque Court	0.64	8.1	Digital literacy, 9; Engagement, 9
	New Year's Party	0.12	9.2	
	Children's Disco	0.37	9.6	
	Parent-Child Fun	0.29	12.4	Digital literacy, 9; Engagement, 11; Trust, 11
False negatives	Boule for all in Telli	0.14	14.2	
Equal shares, `GPT3`	Intergenerational Project	0.52	9.5	Digital literacy, 10; Engagement, 11; Trust, 11
	Open Closet	0.73	10.7	
	Long Table Festival	0.81	7.2	Digital literacy, 7; Engagement, 8
	Let's Play Football	0.62	11.5	
	Open Children's Studio	0.75	2.8	
False positives	Seniors Gathering 70+	0.29	15.2	Digital literacy, 14; Trust, 15
Equal shares, `GPT3`	Petanque Court	0.52	8.8	Engagement, 8
	New Year's Party	0.73	10.1	
	Children's Disco	0.62	7.2	Digital literacy, 7; Engagement, 6; Trust, 6
	Parent-Child Fun	0.75	3.4	

S3.2 Fairness in machine learning architectures

In this section, we discuss the approaches adopted to reduce prediction bias in our machine learning framework, which can arise due to sensitive personal traits such as gender, age, education, and household size [19]. To reduce the impact of the bias from these traits, we augment the approaches suggested by Johnson et al. [19] and formulate an approach based on hyperparmeter optimization and synthetic minority oversampling [5].

The voter data collected through the field study is first analyzed for unequal distributions. We observe that the distributions are quite balanced for gender, age groups, and household size, but unbalanced for political

Table S12: **Consistency recovery by abstaining models is more salient for true positives (1.66 vs. 1.0 for true negatives), whereas in random control populations, it favors true negatives (2.27 vs. 1.89 for true positives).** The recovery for abstaining and control populations (randomly sampled 40 times based on the size of the abstaining group) is analyzed across false negatives, false positives, and different aggregation methods. Recovery is evaluated both for all instances and specifically for cases where project changes occur.

	**Digital literacy # projects**	**Control [digital literacy] Avg. projects %**	**Engagement # projects**	**Control [engagement] Avg. projects %**	**Trust # projects**	**Control [trust] Avg. projects %**
All instances						
Equal shares [abstaining: false negatives; AI: true positives]	1	1.81	2	1.36	3	2.04
Utilitarian greedy [abstaining: false negatives; AI: true positives]	2	2.43	0	1.36	2	2.31
Equal shares [abstaining: false positives; AI: true negatives]	2	2.49	0	1.31	0	2.74
Utilitarian greedy [abstaining: false positives; AI: true negatives]	1	2.44	2	1.98	1	2.67
Equal shares [all additions and removals]	3	2.15	2	1.34	3	2.39
Utilitarian greedy [all additions and removals]	3	2.43	2	1.67	3	2.49
Instances where project changes occur						
Equal shares [abstaining: false negatives; AI: true positives]	1	2.01	2	1.55	3	2.33
Utilitarian greedy [abstaining: false negatives; AI: true positives]	2	2.43	0	1.57	2	2.31
Equal shares [abstaining: false positives; AI: true negatives]	2	2.54	0	1.31	0	2.81
Utilitarian greedy [abstaining: false positives; AI: true negatives]	1	2.44	2	1.98	1	2.67
Equal shares [all additions and removals]	3	2.27	2	1.43	3	2.57
Utilitarian greedy [all additions and removals]	3	2.43	2	1.77	3	2.49

Table S13: **Collective consistency recovery is higher through AI representation in large districts such as Telli, Zelgli, Schachen, and Innenstadt, where at least 25% of the 33 proposed projects originate. Additionally, Altstadt and Scheibenschachen, where more than 30% of the proposed projects are up for voting, also exhibit positive consistency recovery with AI representation.** The consistency recovery is calculated based on the district-specific abstaining voters, adjusted by subtracting the recovery observed in randomly sampled voters of equivalent size under a scenario of 100% AI representation. The reported recovery figures pertain to `GPT3.5`, which demonstrates, on average, 18.2% higher representation than `Llama3-8B` and 20.14% higher than `GPT3`.

	Consistency recovery	
**District**	Equal shares	Utilitarian greedy
Alstadt	6.11	6.51
Ausserfield	8.89	0.91
Binzenhof	-1.55	-8.03
Damn	8.21	-17.21
Goldern	5.16	-3.48
Gonhard	13.22	2.43
Hinterdorf	-0.9	3.48
Hungerberg	7.76	1.31
Innenstadt	8.94	10.33
Rossligut	2.28	-0.40
Schachen	6.14	-7.98
Scheibenschachen	9.94	6.54
Seibenmatten	4.78	-3.31
Torfeld Nod	3.21	-2.18
Torfeld Sud	4.88	3.01
Telli	10.34	4.85
Zelgi	22.22	2.84

Table S14: Comparison of Human, `GPT3.5` representation, `GPT 4-o Mini` representation, and and the actual Pre-election predictions (US National elections 2012–2020): % of votes in favor of each candidate shown. The AI representation is emulated for 100% of the sampled population. Candidates 1 and 2 are the electoral candidates contesting the election.

**Year**	**Human**	`GPT3.5`	`GPT 4-o Mini`	**Pre-Elections**
**2012**				
Candidate 1	55.25%	61.49%	59.14%	48.80%
Candidate 2	37.45%	31.21%	33.55%	48.10%
**2016**				
Candidate 1	45.70%	50.92%	48.11%	43.60%
Candidate 2	41.73%	36.51%	39.32%	48.60%
**2020**				
Candidate 1	53.31%	51.47%	50.59%	51.30%
Candidate 2	37.33%	39.18%	45.30%	46.80%

orientation and basic education.

As an example, around 78.3% of the participants are aligned with left-political beliefs, and 66.7% of the participants are at the highest and second highest levels of education for the actual City Idea voting dataset. We mark the data corresponding to individuals with left political orientation as a privileged group and with right political orientation as a non-privileged group. The same technique is applied to segregate high and low education levels. We randomly sample data separately from these groups, keeping the sample sizes equal, and train a decision tree model to predict the independent variable. We repeat this process for a fixed number of iterations as a stopping criterion and select the final model that achieves the highest recall and the lowest average odds difference [5].

Recall is calculated as $TP/(TP + FN)$ where TP is the true positive, FP is the false positive, TN is the true negative, and FN is the false negative. The average odds difference is calculated as the average difference in the false positive rates and true positive rates for the privileged and non-privileged groups. The false positive rate (FPR) is defined as FPR $= FP/(TP + FN)$, and the true positive rate (TPR) is defined as TPR $= TP/(FP + TN)$ [5].

Apart from addressing the biases for sensitive personal traits, the datasets are also finally checked for a class-wise imbalance, and synthetic minority oversampling [5] is applied for the classes that still remain a minority. This process is helpful for the actual City Idea voting dataset where the number of unique classes is over 25, and even after mitigating the possible biases in the protected variables using oversampling, some classes remain a minority, which can impact the overall prediction capability of the model [6].

S3.3 Incremental prediction of AI choice consistency based on personal human traits groups

In the machine learning architecture, personal human traits are used as features, serving as independent variables, while the consistency gain of voters belonging to the abstaining group is treated as the dependent variable. We have experimented with different supervised machine learning models, including decision trees [12], support vector machines [24], and multilayer perceptrons [35], which do not have long term memory, as well as recurrent neural networks [25], which have long term memory and process and learn information in short interrelated sequences. This capacity of recurrent neural networks to store and remember interpretations from sets of sequences that correspond to groups of perusal human traits helps to mimic human decision making, which is a function of the traits related to socio-demographic characteristics, preferences, political inclination, etc. [9]. Hence among the machine learning models, recurrent neural networks provide the best

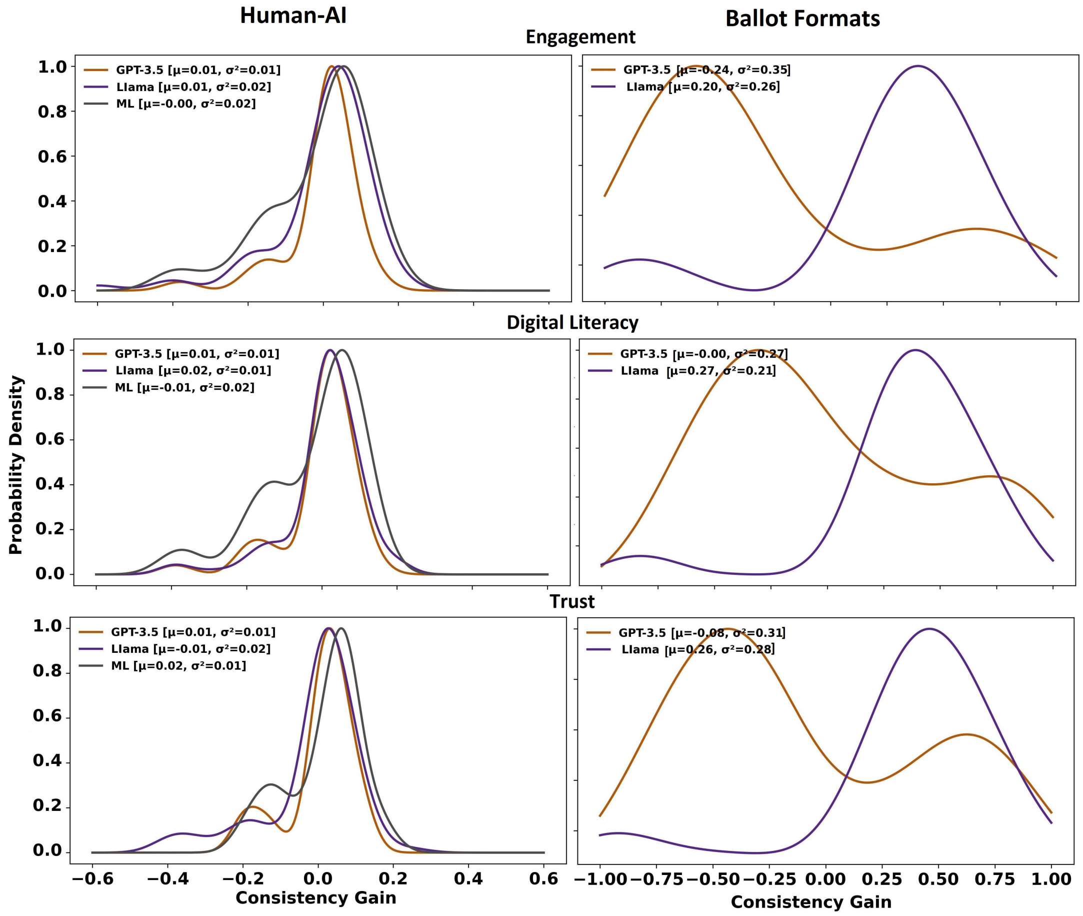

Figure S11: **The divergence in AI choices for abstaining voters, relative to the random baseline population, tends to be neutral, with a slight inclination towards gain.** The AI representation of three abstaining models (low engagement, trust, digital literacy) is evaluated for accuracy against human choices and transitivity across ballot formats for each voter by comparison with random voters. For voters in the abstaining group, the average difference in human–AI accuracy or transitivity between ballot formats is computed by randomly sampling voters, using sample sizes of 20, 30, and 40.

prediction performance.

Consequently, predicting consistency gain / loss becomes a joint probability distribution function ($\mathbb{P}$) of the personal human traits of voters:

$$\mathbb{P}(\text{ballot}) = \mathbb{P}(\text{socio-demographics}) \cdot \mathbb{P}(\text{political interests}) \cdot \mathbb{P}(\text{project preferences}) \cdot \mathbb{P}(\text{outcome expectations})$$

We further test recurrent neural networks with all subsets of the personal human trait groups and hyperparameters, and we observe that holistic integration of all groups (see Table S15) provides the best performance. The performance of consistency gain prediction for the abstaining groups and the entire population is enumerated in Table S16, considering all personal trait groups and recurrent neural networks. The datasets used are obtained from actual and survey voting of the City Idea campaign and the US elections. Our findings indicate that consistency gains can be more accurately predicted for abstaining groups compared to the overall

population.

Table S15: **Using all the personal human traits as features helps in achieving the optimum prediction performance of consistencies of AI choices.** Recurrent Neural Networks to predict (i) the consistency difference between the three abstaining models and their random control and (ii) the (in)consistency of AI representation and transitivity for the whole population. For each dataset, the prediction metrics shown are averaged across both experiments for the datasets for the abstaining groups and the baseline. *Parameters of the best model extracted from hyperparameter tuning*: dense layer of 16 neurons; leaky Relu activation function; categorical cross-entropy loss; adam optimiser; synthetic minority oversampling technique to increase 20% data for all classes; iterations: 600.

**Personal Human Traits**	**Model**	**Survey voting**		**Actual voting**	
		**F1-score**	**Accuracy**	**F1-score**	**Accuracy**
All traits	`Llama3-8B`	0.830	0.836	0.816	0.819
	`GPT3`	0.820	0.799	0.811	0.818
	`GPT3.5`	0.845	0.838	0.821	0.825
Socio-demographics and political interests	`Llama3-8B`	0.610	0.618	0.616	0.613
	`GPT3`	0.642	0.640	0.635	0.634
	`GPT3.5`	0.612	0.602	0.616	0.603
Socio-demographics, project preferences and outcome expectations	`Llama3-8B`	0.714	0.719	0.698	0.700
	`GPT3`	0.753	0.760	0.712	0.721
	`GPT3.5`	0.687	0.679	0.721	0.725
Socio-demographics, political interests and outcome expectations	`Llama3-8B`	0.661	0.657	Only survey voting	
	`GPT3`	0.685	0.688		
	`GPT3.5`	0.709	0.715		
Socio-demographics, political interests and project preferences	`Llama3-8B`	0.672	0.689	only survey voting	
	`GPT3`	0.646	0.656		
	`GPT3.5`	0.6652	0.663		

Table S16: **The performance statistics for every abstaining group and baseline for predicting the consistency of AI choices with respect to human choices and within ballot formats.** The F1-Score reported is based on the experiment conducted using all the traits using recurrent neural networks - dense layer of 16 neurons; leaky Relu activation function; categorical cross-entropy loss; adam optimiser; synthetic minority oversampling technique to increase 20% data for all classes; epoch: 600.

**Human-AI (F1-Scores)**																								
	**City Idea [Actual]**												**City Idea [Survey]**											
**Ballots**	**Engagement**			**Digital literacy**			**Trust**			**Baseline**			**Engagement**			**Digital literacy**			**Trust**			**Baseline**		
Score	0.86	0.83	0.86	0.88	0.88	0.88	0.86	0.88	0.87	0.78	0.78	0.83	0.83	0.83	0.82	0.87	0.88	0.88	0.85	0.84	0.86	0.74	0.71	0.74
Approval	0.85	0.85	0.87	0.88	0.9	0.87	0.89	0.86	0.89	0.79	0.76	0.81	0.82	0.82	0.83	0.86	0.84	0.86	0.85	0.83	0.85	0.73	0.74	0.75
**Within Ballot Formats**																								
Single Choice - Score	0.83	0.83	0.82	0.87	0.83	0.83	0.85	0.84	0.86	0.74	0.71	0.74	0.81	0.83	0.84	0.87	0.88	0.88	0.85	0.84	0.88	0.74	0.73	0.76
Single Choice - Approval	0.83	0.83	0.81	0.86	0.82	0.85	0.84	0.81	0.85	0.73	0.71	0.73	0.83	0.84	0.84	0.86	0.86	0.85	0.84	0.83	0.86	0.75	0.73	0.73
**US Elections -** `GPT3.5` **= 0.89;** `Llama3-8B` **= 0.86; ML= 0.89 (averged over all three years) for single choice ballots**																								

S3.4 Explainability of choices

We causally analyze personal human traits and their contribution to consistency for each voter at the individual level using local explainable AI methods such as Shapley Additive Explanations (SHAP) and Local Interpretable Model Agnostic Explanations (LIME) [16], along with a relative analysis across all voters using a global feature ablation study [18]. The findings using SHAP and LIME methods are outlined in Figures 5 (main paper), S12, S13, S14, S15 and S16 for all types of ballots. The observations from the feature ablation study are detailed in Table S17. The mapping of relevant personal human traits to cognitive biases is discussed for score or cumulative ballots in Figure 5 (main paper) and for approval ballots in Table S18.

Table S17: **Preference for fairness and welfare positively contribute to the Human-AI consistencies for voters with low digital literacy and low trust, respectively. preference for family projects positively contributes to within-ballot format consistencies for all three abstaining populations.** the traits are tested for their relative importance using feature ablation methods [18] to extract the mean decrease in accuracy after removing them from the model. the top 3 important features with high errors and the bottom 2 features with the least errors are noted.

Model	Top 1	Top 2	Top 3	Bottom 1	Bottom 2
**City Idea [Actual] - human AI consistency**					
Engagement	Public transit (0.17)	Self benefit (0.15)	Children (0.10)	Interests in politics (-0.003)	Sports (-0.0025)
Digital literacy	Families (0.16)	Fairness (0.10)	Children (0.08)	Trust democracy (-0.005)	Culture (0.0010)
Trust	Welfare (0.14)	Fairness (0.12)	Health (0.11)	Urban greenery (0.002)	Sports (-0.001)
**City Idea [Actual] - within ballot formats**					
Engagement	Families (0.09)	Education (0.07)	Welfare (0.07)	Interest in politics (-0.002)	Public space (0.004)
Digital literacy	Education (0.10)	Families (0.05)	Health (0.04)	Interest in politics (-0.002)	Public space (-0.004)
Trust	Welfare (0.12)	Health (0.11)	Families (0.09)	Fairness (-0.003)	Public space (-0.001)
**City Idea [Survey] - human AI consistency**					
Engagement	Public transit (0.20)	Self benefit (0.19)	Health (0.18)	Interest in politics (-0.004)	Urban greenery (-0.003)
Digital literacy	City benefit (0.18)	Education (0.13)	Health (0.11)	Public space (0.005)	Urban greenery (-0.001)
Trust	Welfare (0.18)	Health (0.17)	Interest in politics (0.16)	Elderly (-0.0012)	Environment (0.003)
**City Idea [Survey] - within ballot format**					
Engagement	Public space (0.19)	Environment (0.17)	Families (0.16)	Elderly (-0.005)	Interests in politics (-0.001)
Digital literacy	Families (0.19)	Elderly (0.15)	Welfare (0.15)	Public space (0.006)	Interests in politics (0.006)
Trust	Families (0.15)	Health (0.14)	Fairness (0.14)	Interests in politics (0.003)	Sports (0.002)

Table S18: **Compared to an arbitrary abstaining voter, those with low engagement and digital literacy exhibit characteristics that explain the consistency of human-AI representation and ballot formats, for instance no interest in politics and support to family initiatives corresponding to unconscious and surrogation biases.** The significant biases observed in approval ballots across all abstention models, based on both survey data and actual City Idea campaign, have been aggregated using relative importance scores and significance values. The explainable AI methods used are Shapley Additive Explanations (SHAP) and Local Interpretable Model Agnostic Explanations (LIME) [16].

**Features**	**Relative importance [%]**	**p-value**	**Explainable AI method**	**Type of consistency**	**Ballot formats**	**[Abstaining models]**
Not interested in politics	14.2	0.031	SHAP	Human - AI	Approval	[Engagement]
Not interested in politics	12.4	0.024	LIME	Human - AI	Approval	[Engagement]
Interested in self benefit	16.7	0.038	SHAP	Human - AI	Approval	[Engagement]
Interested in self benefit	17.3	0.041	LIME	Human - AI	Approval	[Engagement]
Support to family initiatives	19.2	0.002	SHAP	Ballot formats	Single choice - approval	[Engagement]
Support to family initiatives	18.8	0.045	LIME	Ballot formats	Single choice - approval	[Engagement]
Support to city benefits	13.4	0.003	SHAP	Human - AI	Approval	[Digital literacy]
Support to city benefits	14.6	0.004	LIME	Human - AI	Approval	[Digital literacy]
Support to health initiatives	12.3	0.003	SHAP	Ballot formats	Single choice - approval	[Digital literacy]

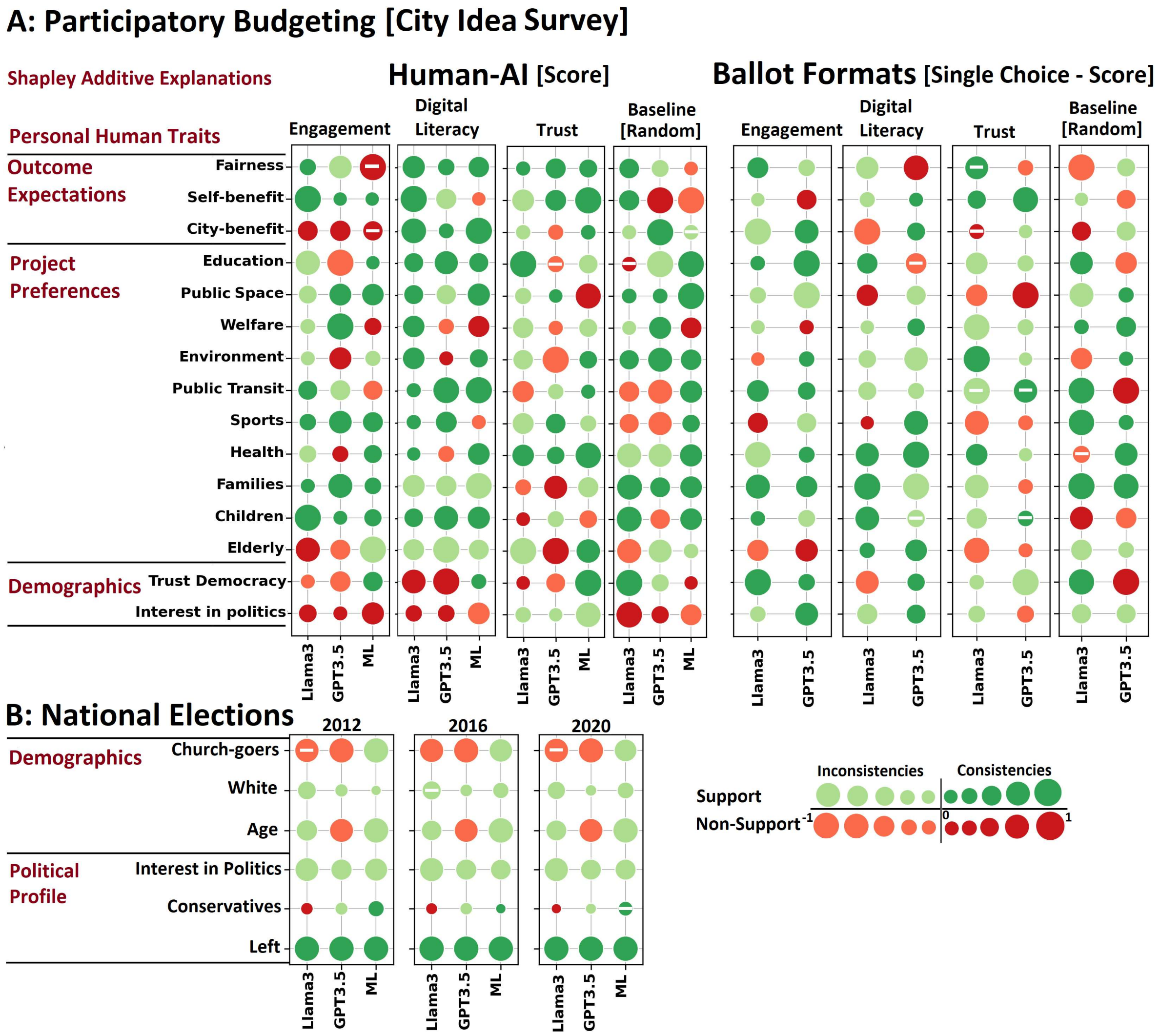

Figure S12: **Compared to an arbitrary abstaining voter, those with low engagement and digital literacy exhibit characteristics that explain the consistency of human-AI representation and ballot formats, for instance, no interest in politics and support to education/health projects related to unconscious and surrogation biases. Time discounting, affect and conformity biases, such as preference for public space and environmental projects as well as support to families contribute to the consistency of human-AI choice. For US elections, unconscious bias such as political beliefs positively impacts the human-AI consistency.** The relative importance of the personal human traits (y-axis) are shown for the (A) survey participatory budgeting campaign of City Idea and the (B) US Elections using the size of the bubbles and it is calculated using Shapley Additive Explanations. The AI representation is shown for `GPT3.5` and `Llama3-8B` (Llama) along with the predictive model (ML) (x-axis). The consistency of human-AI representation (score ballots) and ballot formats (single choice vs. score) is assessed. For each of these, the personal human traits explain the following: (i) The consistency difference between the three abstaining models and their random control. (ii) The (in)consistency of AI representation and transitivity for the whole population. The '-' sign indicates non-significant values (p>0.05).

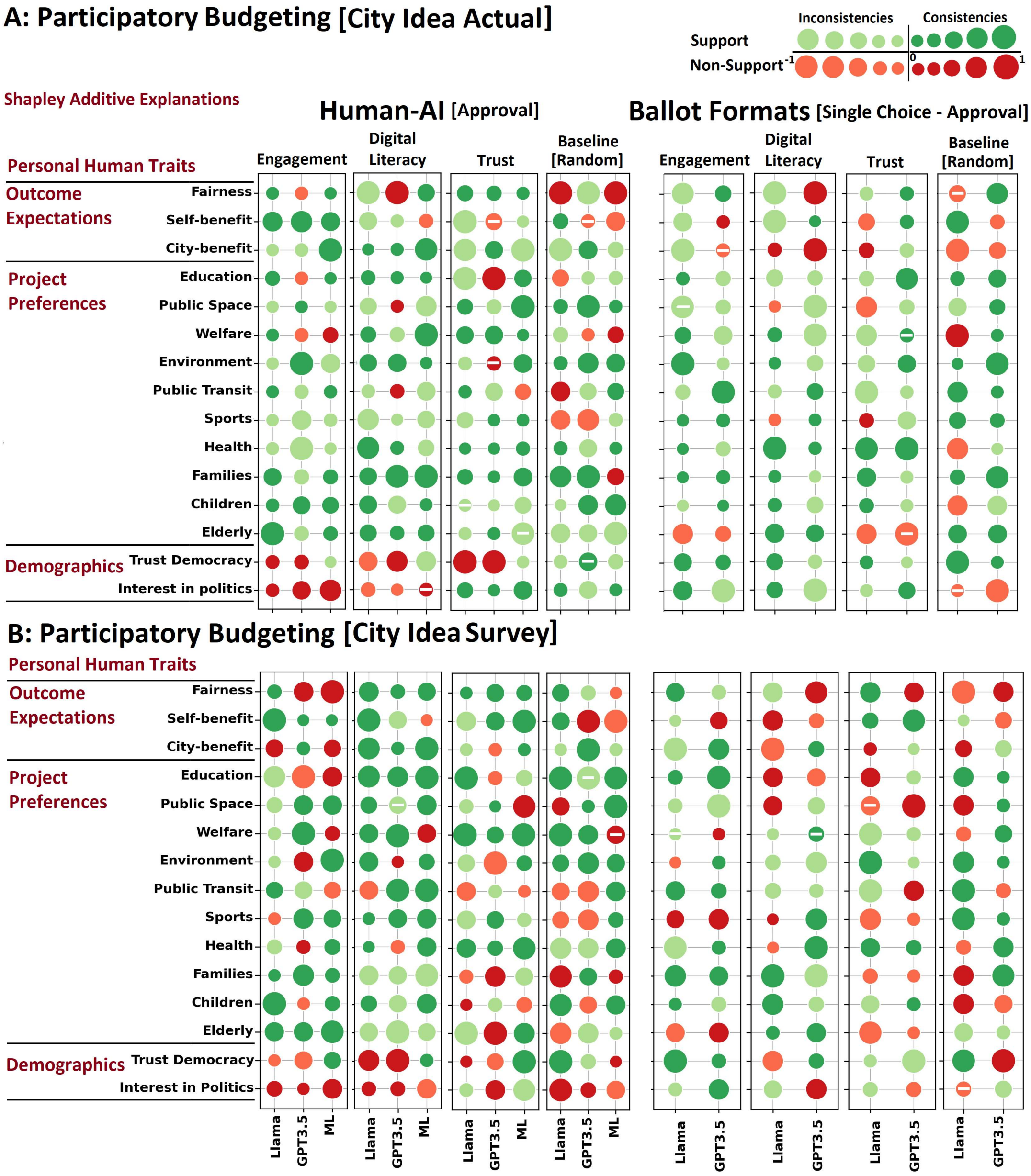

Figure S13: **Voters with low engagement and digital literacy exhibit traits that explain ballot format consistency and human-AI choices, such as no interest in politics or supporting initiatives with citywide benefits related to unconscious and altruism bias.** The relative importance of the personal human traits (y-axis) for the (A) actual and (B) survey participatory budgeting campaign of City Idea for `GPT3.5` and `Llama3-8B` (Llama) along with the predictive model (ML) (x-axis) are depicted by the size of the bubbles and it is calculated using Shapley Additive Explanations. The consistency of human-AI representation (approval ballots) and ballot formats (single choice vs. approval is assessed. For each of these, the personal human traits explain the following: (i) The consistency difference between the three abstaining models and their random control. (ii) The (in)consistency of AI representation and transitivity for the whole population. The '-' sign indicates non-significant values (p>0.05).

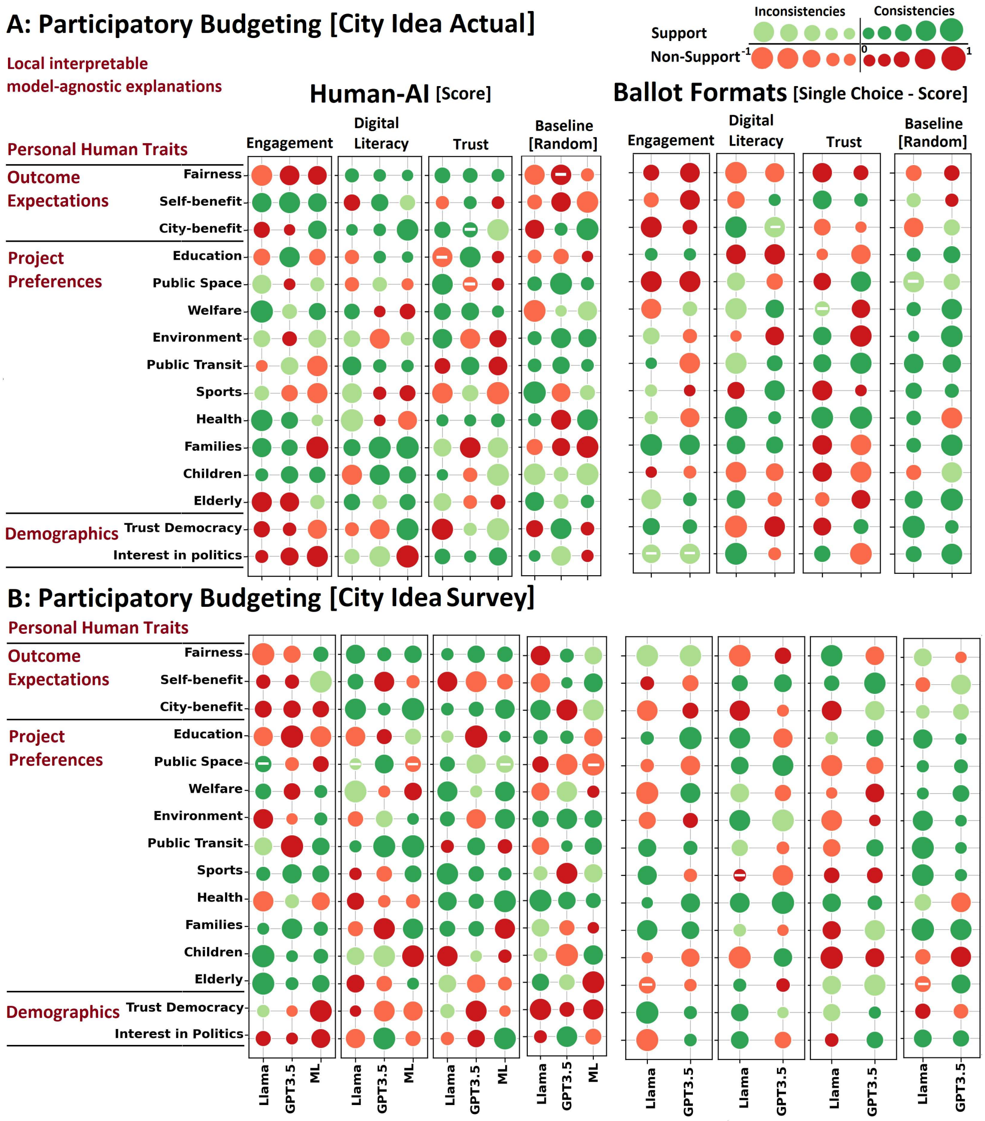

Figure S14: **Voters with low engagement and digital literacy exhibit traits that explain human-AI choices and ballot format consistency, such as no interest in politics or supporting health initiatives related to unconscious and surrogation bias.** The relative importance of the personal human traits (y-axis) for the (A) actual and (B) survey participatory budgeting campaign of City Idea for `GPT3.5` and `Llama3-8B` (Llama) along with the predictive model (ML) (x-axis) are depicted by the size of the bubbles and it is calculated using Local Interpretable Model-agnostic Explanations. The consistency of human-AI representation (score / cumulative ballots) and ballot formats (single choice vs. score cumulative) is assessed. For each of these, the personal human traits explain the following: (i) The consistency difference between the three abstaining models and their random control. (ii) The (in)consistency of AI representation and transitivity for the whole population. The '-' sign indicates non-significant values (p>0.05).

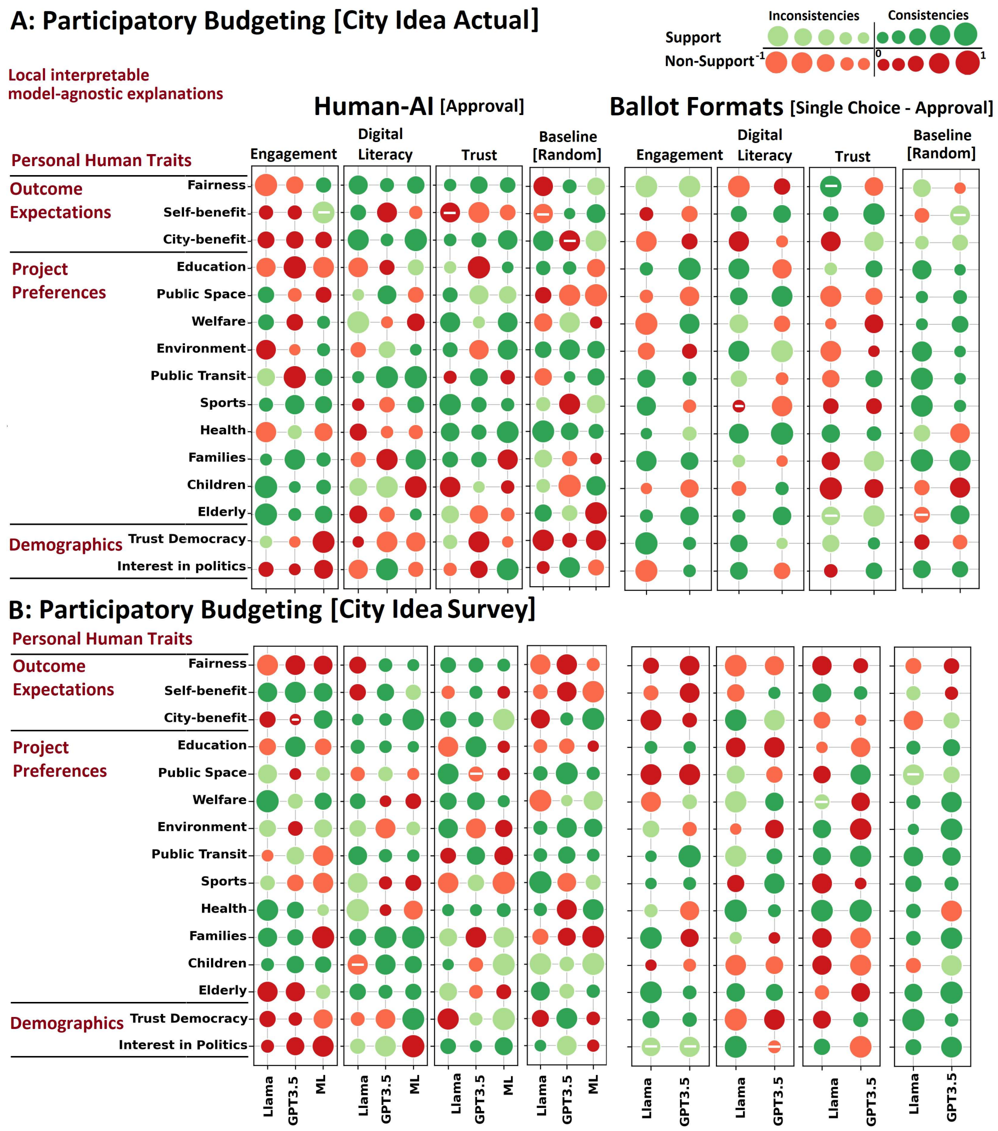

Figure S15: **Voters with low engagement and digital literacy exhibit traits that explain human-AI choices and ballot format consistency, such as no interest in politics or supporting health initiatives related to unconscious and surrogation bias.** The relative importance of the personal human traits (y-axis) for the (A) actual and (B) survey participatory budgeting campaign of City Idea for `GPT3.5` and `Llama3-8B` (Llama) along with the predictive model (ML) (x-axis) are depicted by the size of the bubbles and it is calculated using Local Interpretable Model-agnostic Explanations. The consistency of human-AI representation (approval ballots) and ballot formats (single choice vs. approval) is assessed. For each of these, the personal human traits explain the following: (i) The consistency difference between the three abstaining models and their random control. (ii) The (in)consistency of AI representation and transitivity for the whole population. The '-' sign indicates non-significant values (p>0.05).

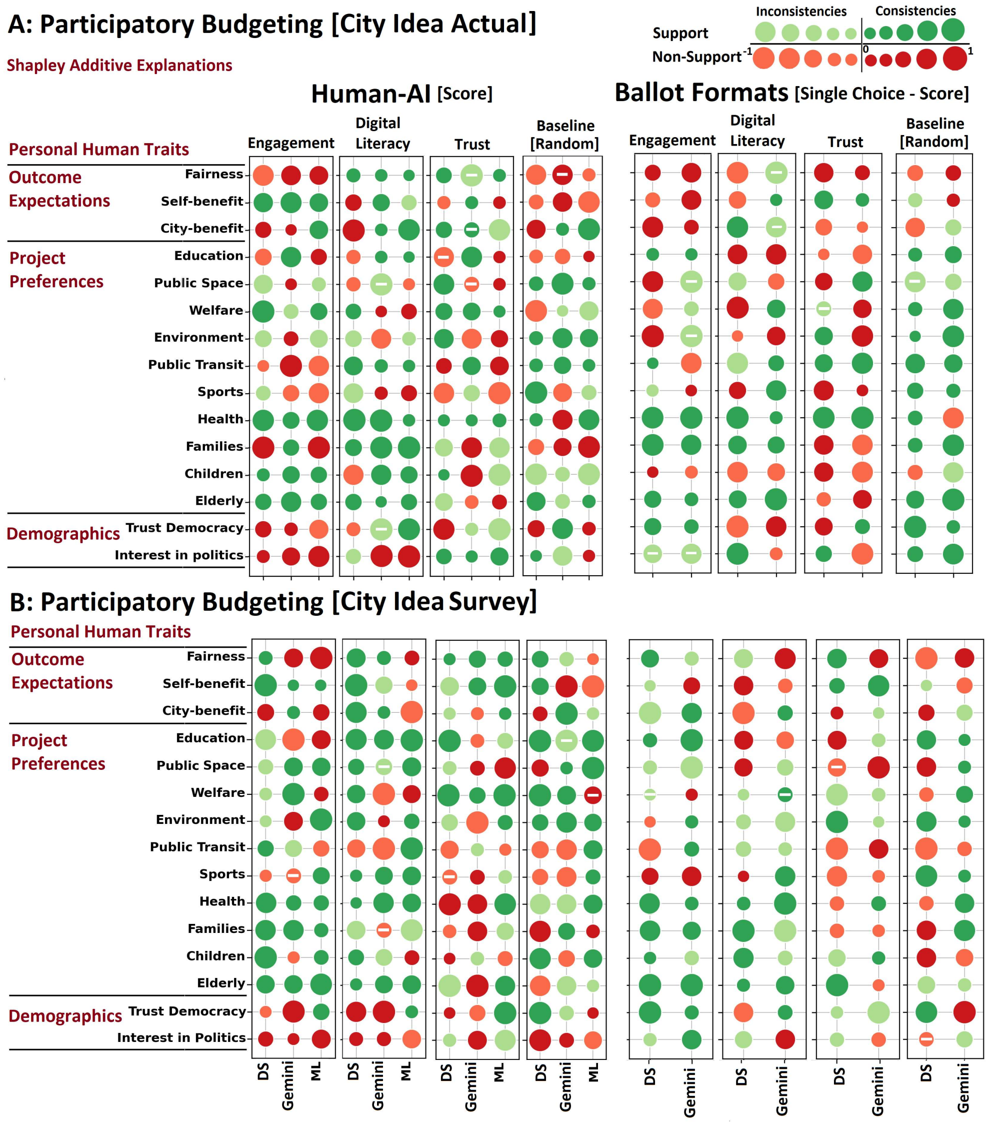

Figure S16: **Voters with low engagement and digital literacy exhibit traits that explain human-AI choices and ballot format consistency, such supporting health and elderly initiatives related to surrogation bias.** The relative importance of the personal human traits (y-axis) for the (A) actual and (B) survey participatory budgeting campaign of City Idea for `Gemini 1.5 Flash` (Gemini) and `Deepseek R1` (DS) along with the predictive model (ML) (x-axis) are depicted by the size of the bubbles and it is calculated using Shapley Additive Explanations. The consistency of human-AI representation (score ballots) and ballot formats (single choice vs. score) is assessed. For each of these, the personal human traits explain the following: (i) The consistency difference between the three abstaining models and their random control. (ii) The (in)consistency of AI representation and transitivity for the whole population. The '-' sign indicates non-significant values ($p>0.05$).

References

[1] Chiara Acciarini, Federica Brunetta, and Paolo Boccardelli. Cognitive biases and decision-making strategies in times of change: a systematic literature review. *Management Decision*, 59(3):638–652, 2021.

[2] Elliot Anshelevich, Onkar Bhardwaj, Edith Elkind, John Postl, and Piotr Skowron. Approximating optimal social choice under metric preferences. *Artificial Intelligence*, 264:27–51, 2018.

[3] Lisa P Argyle, Ethan C Busby, Nancy Fulda, Joshua R Gubler, Christopher Rytting, and David Wingate. Out of one, many: Using language models to simulate human samples. *Political Analysis*, 31(3):337–351, 2023.

[4] Markus Brill, Rupert Freeman, Svante Janson, and Martin Lackner. Phragmén's voting methods and justified representation. *Mathematical programming*, 203(1):47–76, 2024.

[5] Joymallya Chakraborty, Suvodeep Majumder, and Tim Menzies. Bias in machine learning software: Why? how? what to do? In *European software engineering conference and symposium on the foundations of software engineering, ACM*, pages 429–440, 2021.

[6] Joymallya Chakraborty, Suvodeep Majumder, Zhe Yu, and Tim Menzies. Fairway: a way to build fair ML software. In *European software engineering conference and symposium on the foundations of software engineering, ACM*, pages 654–665, 2020.

[7] Keyu Chen and Shiliang Sun. CP-Rec: contextual prompting for conversational recommender systems. In *Conference on Artificial Intelligence, AAAI*, number 11, pages 12635–12643, 2023.

[8] Ruizhe Chen, Yichen Li, Zikai Xiao, and Zuozhu Liu. Large language model bias mitigation from the perspective of knowledge editing. *arXiv preprint arXiv:2405.09341*, 2024.

[9] Celine Colombo and Marco R Steenbergen. *Heuristics and biases in political decision making.* Oxford Research Encyclopedia of Politics, 2020.

[10] Richard Conniff. Using peer pressure as a tool to promote greener choices. *Yale Environment*, 360:1–5, 2009.

[11] Barbara Culiberg and Leila Elgaaied-Gambier. Going green to fit in–understanding the impact of social norms on pro-environmental behaviour, a cross-cultural approach. *International journal of consumer studies*, 40(2):179–185, 2016.

[12] Barry De Ville. Decision trees. *Wiley Interdisciplinary Reviews: Computational Statistics*, 5(6):448–455, 2013.

[13] Roberto Falanga, Jessica Verheij, and Olivia Bina. Green (er) cities and their citizens: insights from the participatory budget of lisbon. *Sustainability*, 13(15):8243, 2021.

[14] Ernst Fehr and Klaus M Schmidt. The economics of fairness, reciprocity and altruism–experimental evidence and new theories. *Handbook of the economics of giving, altruism and reciprocity*, 1:615–691, 2006.

[15] Tracy Fiander Trask. *The role of affect and cognition in predicting attitudes toward the elderly.* PhD thesis, Memorial University of Newfoundland, 1999.

[16] Kary Främling, Marcus Westberg, Martin Jullum, Manik Madhikermi, and Avleen Malhi. Comparison of contextual importance and utility with lime and shapley values. In *International Workshop on Explainable, Transparent Autonomous Agents and Multi-Agent Systems, Springer*, pages 39–54. Springer, 2021.

[17] Christian Gollier. *Pricing the future: The economics of discounting and sustainable development.* Princeton University Press, Princeton, NJ, USA, 2011.

[18] Isha Hameed, Samuel Sharpe, Daniel Barcklow, Justin Au-Yeung, Sahil Verma, Jocelyn Huang, Brian Barr, and C Bayan Bruss. BASED-XAI: Breaking ablation studies down for explainable artificial intelligence. *arXiv preprint arXiv:2207.05566*, 2022.

[19] Brittany Johnson, Jesse Bartola, Rico Angell, Katherine Keith, Sam Witty, Stephen J Giguere, and Yuriy Brun. Fairkit, fairkit, on the wall, who's the fairest of them all? supporting data scientists in training fair models. *arXiv preprint arXiv:2012.09951*, 2020.

[20] Johan Korteling, Geerte L Paradies, Josephine P Sassen-van Meer, et al. Cognitive bias and how to improve sustainable decision making. *Frontiers in Psychology*, 14:1129835, 2023.

[21] James H Kuklinski and Buddy Peyton. Belief systems and political decision making. *The Oxford Handbook of Political Behavior*, 2007.

[22] Konrad Kułakowski, Jiri Mazurek, and Michał Strada. On the similarity between ranking vectors in the pairwise comparison method. *Journal of the Operational Research Society*, 73(9):2080–2089, 2022.

[23] Sajan Maharjan, Srijoni Majumdar, and Evangelos Pournaras. Fair voting outcomes with impact and novelty compromises? unravelling biases in electing participatory budgeting winners. *Philosophical Transactions A*, 382(2285):20240096, 2024.

[24] Alessia Mammone, Marco Turchi, and Nello Cristianini. Support vector machines. *Wiley Interdisciplinary Reviews: Computational Statistics*, 1(3):283–289, 2009.

[25] Danilo Mandic and Jonathon Chambers. *Recurrent neural networks for prediction: learning algorithms, architectures and stability.* Wiley, 2001.

[26] Ggaliwango Marvin, Nakayiza Hellen, Daudi Jjingo, and Joyce Nakatumba-Nabende. Prompt engineering in large language models. In *International conference on data intelligence and cognitive informatics, Springer*, pages 387–402. Springer, 2023.

[27] Usman Naseem, Imran Razzak, Shah Khalid Khan, and Mukesh Prasad. A comprehensive survey on word representation models: From classical to state-of-the-art word representation language models. *Transactions on Asian and Low-Resource Language Information Processing*, 20(5):1–35, 2021.

[28] Carlos Navarrete, Mariana Macedo, Rachael Colley, Jingling Zhang, Nicole Ferrada, Maria Eduarda Mello, Rodrigo Lira, Carmelo Bastos-Filho, Umberto Grandi, Jérôme Lang, et al. Understanding political divisiveness using online participation data from the 2022 french and brazilian presidential elections. *Nature Human Behaviour*, 8(1):137–148, 2024.

[29] Jasmin Odermatt, Lea Good, and Mina Najdl. Stadtidee: Partizipatives budget. `https://www.stadtidee.aarau.ch/public/upload/assets/31299/Abschlussbericht%20zur%20Stadtidee%202023-2024_final_neu.pdf`, 2025. [Online; accessed 21-January-2026].

[30] Marco Percoco and Peter Nijkamp. Individuals time preference and social discounting: A survey and a meta-analysis. In *Congress of the European Regional Science Association, ERSA*, number 5, pages 1–36, 2006.

[31] Dominik Peters, Grzegorz Pierczynski, and Piotr Skowron. Proportional participatory budgeting with cardinal utilities. *arXiv preprint arXiv:2008.13276*, pages 2181–2188, 2020.

[32] Dominik Peters, Grzegorz Pierczyński, and Piotr Skowron. Proportional participatory budgeting with additive utilities. *Advances in Neural Information Processing Systems*, 34:12726–12737, 2021.

[33] Yiwei Wang, Yujun Cai, Muhao Chen, Yuxuan Liang, and Bryan Hooi. Primacy effect of chatGPT. *arXiv preprint arXiv:2310.13206*, 2023.

[34] Jason Wei, Xuezhi Wang, Dale Schuurmans, Maarten Bosma, Fei Xia, Ed Chi, Quoc V Le, Denny Zhou, et al. Chain-of-thought prompting elicits reasoning in large language models. *Advances in neural information processing systems*, 35:24824–24837, 2022.

[35] Yu-chen Wu and Jun-wen Feng. Development and application of artificial neural network. *Wireless Personal Communications*, 102(10):1645–1656, 2018.

[36] Yijiong Yu, Huiqiang Jiang, Xufang Luo, Qianhui Wu, Chin-Yew Lin, Dongsheng Li, Yuqing Yang, Yongfeng Huang, and Lili Qiu. Mitigate position bias in large language models via scaling a single dimension. *arXiv preprint arXiv:2406.02536*, 2024.
